# A near-infrared probe for non-invasively monitoring cerebrospinal fluid flow by ^18^F-positron emitting tomography and fluorescence

**DOI:** 10.1186/s13550-020-0609-3

**Published:** 2020-04-16

**Authors:** Hua Guo, Harikrishna Kommidi, Carl C. Lekaye, Jason Koutcher, Martin S. Judenhofer, Simon R. Cherry, Amy P. Wu, Oguz Akin, Mark M. Souweidane, Omer Aras, Zhaohui Zhu, Richard Ting

**Affiliations:** 1 Department of Nuclear Medicine, Chinese Academy of Medical Sciences and Peking Union Medical College Hospital, Beijing, 100730 China; 2 Beijing Key Laboratory of Molecular Targeted Diagnosis and Therapy in Nuclear Medicine, Beijing, 100730 China; 3 Department of Radiology, Molecular Imaging Innovations Institute (MI3), Weill Cornell Medical College, New York, NY 10065 USA; 4 Department of Medical Physics, Memorial Sloan Kettering Cancer Center, New York, NY 10065 USA; 5 Department of Biomedical Engineering, University of California at Davis, Davis, CA 95616 USA; 6 Department of Otolaryngology – Head & Neck Surgery, Northwell Health, Hofstra Northwell School of Medicine, New York, NY 10075 USA; 7 Department of Radiology, Memorial Sloan Kettering Cancer Center, New York, NY 10065 USA; 8 Department of Neurological Surgery, Weill Cornell Medicine, New York, NY 10065 USA

**Keywords:** Positron emission tomography, Fluorescence, Non-invasively, CSF flow, CNS disease

## Abstract

**Purpose:**

Knowing the precise flow of cerebrospinal fluid (CSF) is important in the management of multiple neurological diseases. Technology for non-invasively quantifying CSF flow would allow for precise localization of injury and assist in evaluating the viability of certain devices placed in the central nervous system (CNS).

**Methods:**

We describe a near-infrared fluorescent dye for accurately monitoring CSF flow by positron emission tomography (PET) and fluorescence. IR-783, a commercially available near-infrared dye, was chemically modified and radiolabeled with fluorine-18 to give [^18^F]-IR783-AMBF_3_. [^18^F]-IR783-AMBF_3_ was intrathecally injected into the rat models with normal and aberrant CSF flow and evaluated by the fluorescence and PET/MRI or PET/CT imaging modes.

**Results:**

IR783-AMBF_3_ was clearly distributed in CSF-containing volumes by PET and fluorescence. We compared IR783-AMBF_3_ (fluorescent at 778/793 nm, ex/em) to a shorter-wavelength, fluorescein equivalent (fluorescent at 495/511 nm, ex/em). IR783-AMBF_3_ was superior for its ability to image through blood (hemorrhage) and for imaging CSF-flow, through-skin, in subdural-run lumboperitoneal shunts. IR783-AMBF_3_ was safe under the tested dosage both in vitro and in vivo*.*

**Conclusion:**

The superior imaging properties of IR783-AMBF_3_ could lead to enhanced accuracy in the treatment of patients and would assist surgeons in non-invasively diagnosing diseases of the CNS.

## Introduction

Advances in molecular imaging have enhanced our ability to non-invasively track molecules in patients. Yet, no current single imaging modality is ideal, as each individual imaging modality is limited by unique temporal, spatial, and depth (through-tissue) resolutions [1]. One popular strategy in the development of more universal contrast agents is to combine different imaging modalities with synergistic properties, where one modality will complement the resolution shortcomings of another imaging modality [2]. One good combination is positron emission tomography (PET) and fluorescence imaging. Both ^18^F-PET and fluorescence imaging allow the tracking of small molecules at sub-nanomolar concentration, making them among the most sensitive, and therefore also the potentially least toxic, of available imaging modalities [3–6]. Moreover, while PET emissions can be non-invasively quantitated through a patient’s body, fluorescence imaging is ideal for visualizing submicron, histologic structure in superficial tissue. Therefore, by combining ^18^F-PET and fluorescent imaging modalities within a single molecule, we can transcend the molecular resolution limits of any one imaging modality [2, 7–9].

Damage to the central nervous system (CNS) can significantly reduce a patient’s quality of life, especially if not promptly addressed [10, 11]. Unfortunately, CNS injuries can be difficult to identify, especially in patients who are unconscious or who have spontaneous or deep-tissue cerebrospinal fluid (CSF) leakage, where the anatomical CSF leak source is not obvious. A popular strategy for indicating CNS damage is to introduce a contrast agent into the CNS [12–17], but tracking small molecules deep within the CNS represents a particular challenge for multiple reasons: (1) Toxicity to structures within the CNS result in particularly morbid prognoses; therefore, it is crucial that new contrast be useful at low concentrations to ensure non-toxicity [18]. (2) Dense tissue and the thick bone that shield the CNS can scatter both exciting and emitting photons, thereby reducing the quality of certain imaging modalities significantly. (3) Media such as CSF can move a molecule, thereby quickly reducing the usefulness of slower image acquisition strategies that involve faster scanning or signal averaging [13, 15, 19].

To meet this challenge, we previously developed a fluorescein-based PET/fluorescent probe (Fc-AMBF_3_) for imaging within the CNS [20, 21]. To make this probe compatible with the most current FDA-approved intraoperative robotic systems, which are designed to visualize fluorescent near-infrared dyes (> 600 nm) [22], we further develop a near-infrared dye as a PET/fluorescent probe. In this study, we report the synthesis of a near-infrared dye, IR-783 derivative, IR783-AMBF_3_, a.k.a. (((2-((E)-6'-((E)-2-(3,3-dimethyl-1-(4-sulfobutyl)-3H-indol-1-ium-2-yl)vinyl)-2'-(2-((E)-3,3-dimethyl-1-(4-sulfobutyl)indolin-2-ylidene)ethylidene)-2',3',4',5'-tetrahydro-[1,1'-biphenyl]-4-carboxamido)ethyl)dimethylammonium methyltrifluoroborate. IR783-AMBF_3_ was radiolabeled via isotopic exchange radiolabeling with fluorine-18 to give a near-infrared PET/fluorescent probe. We explored the utility of IR783-AMBF_3_ in multiple rat models bearing normal and aberrant CSF flow. We compared IR783-AMBF_3_ with previously published Fc-AMBF_3_. The ability to image CSF flow in deep tissue and through blood suggests that IR783-AMBF_3_ can be used as a tool for diagnosing CNS diseases that involve CSF flow by both PET and fluorescence imaging.

## Materials and methods

### General synthetic methods

Commercial chemicals and solvents were obtained from suppliers and used as purchased. Endotoxin-free and mycoplasma-free, isotonic sterile 1 × PBS (pH 7.4) was obtained from Corning (cellgro®, Product #21-040-CM). Mass spectra were measured with a Waters Acquity H class HPLC/SQD2 mass spectrometer (UPLC−MS) using Acquity UPLC 1.7 μm C18 100 Å, 2.1 × 50 mm column (186002350), with a 4.0 min 10–90% H_2_O/acetonitrile (ACN) (0.05% TFA) gradient and a flow rate of 0.4 mL/min. Preparative HPLC was performed on an Agilent 1260 series HPLC equipped on a Phenomenex Luna C18(2)100 Å, 250 cm × 21.20 mm i.d. 10 μm reverse phase column (00G4253-P0 AX), with a 40 min 10–80% H_2_O/ACN (0.05% TFA) gradient and a flow rate of 12 mL/min. Nuclear magnetic resonance (NMR) spectra were recorded in a deuterated solvent with a 500-MHz Bruker spectrometer. Fluoride-18 concentration was done in a Thermo Scientific 5-mL vial (React-Vial no. 13223). Purity of radiolabeling (≥ 95%) was verified on a Varian reverse phase HPLC, using a Waters SunfireTM C18 3.5 μm, 4.6 mm × 50 mm column (186002551), an attached radiodetector, and a 10–90% H_2_O/ACN (0.05% TFA) elution gradient with a flow rate of 2 mL/min.

### Chemical synthesis

**Scheme 1:**
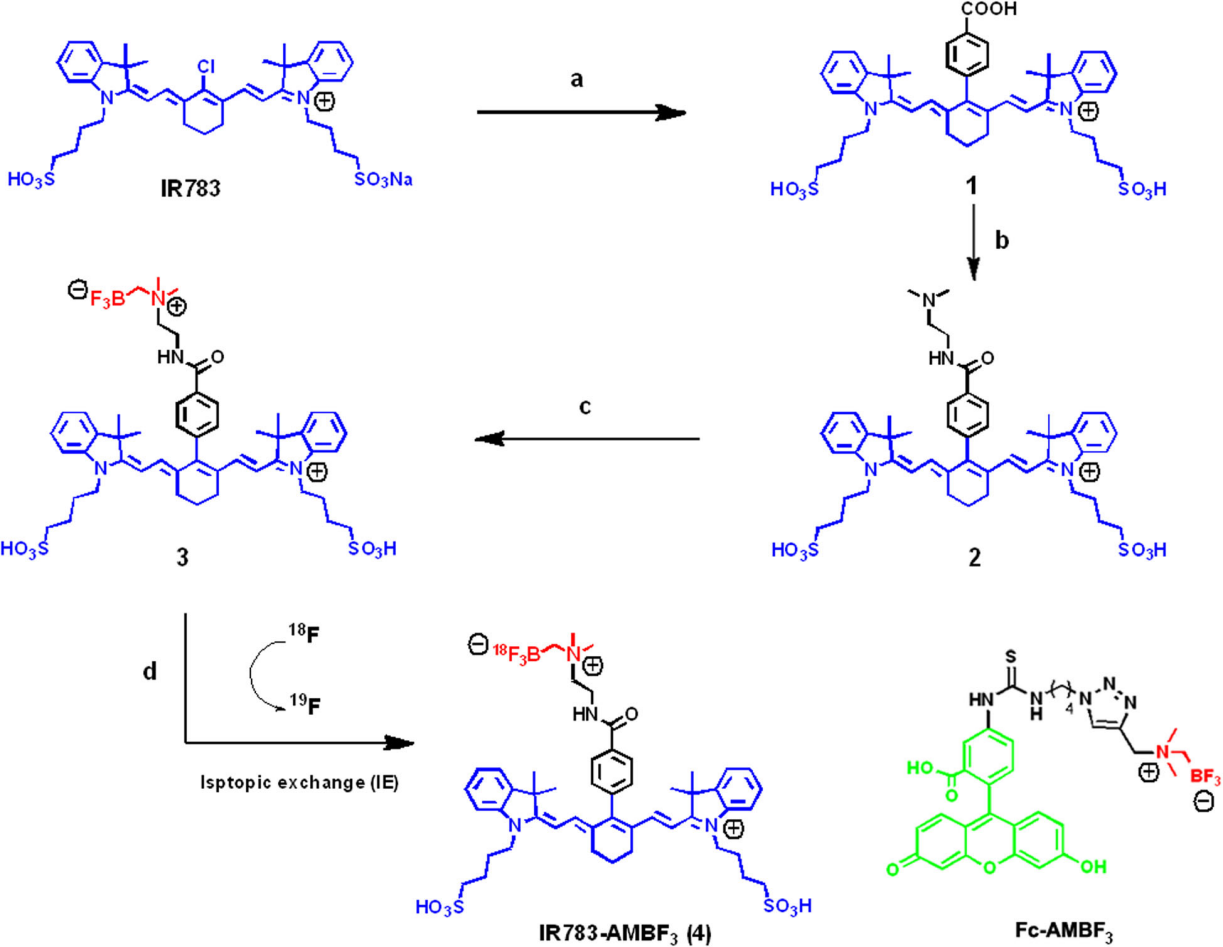
Synthesis of IR783-AMBF_3_. (a) 1.2 eq. 4-Carboxyphenylboronic acid, 0.1 eq. Pd(PPh_3_)_4_, H_2_O/DMF (10:1), 10 h, 100 °C. (b) 1.2 eq. *N*,*N*-dimethylethyl amine, 1.5 eq. HOBt, 1.5 eq. Py, 3.0 eq. EDC. HCl, DMF, 5 h, RT. (c) (i) 2.0 eq. Bromomethylboronic acid pinacol ester, 1.0 eq. *N*,*N*-diisopropylethylamine, DMF, 3 h, RT. (ii) 2.0 M KHF_2_, 1.0 M HCl, 0.5 h, RT. (d) Radiolabeling 1 M Pyridazine-HCl, 40 mCi aqueous [^18^F]-fluoride ion (Specific concentration of > 1.5 Ci/mL), 80 °C, 25 min. Bottom middle structure: IR783-AMBF_3_, a derivative of IR783; Bottom right structure: Fc-AMBF_3_, a previously published fluorescein derivative, to be compared with IR783-AMBF_3_

Reagents and conditions used to synthesize [^18^F]-IR783-AMBF_3_ are described in Scheme 1. IR-783 was coupled to 4-carboxy phenylboronic acid via a Suzuki reaction catalyzed with a trace amount of Pd(PPh_3_)_4_. The obtained acid, **1**, was then reacted with an equivalent of *N*,*N*-dimethyl ethylamine in the presence of 1-ethyl-3-(3-dimethylaminopropyl)carbodiimide hydrochloride (EDCI) and HOBt for 5 h at room temperature. This gave the IR-783-tertiary amine, **2**. Alkylation of **2** with bromo methylboronate, and potassium hydrogen fluoride workup gave the desired ammonium trifluoroborate (IR783-AMBF_3_), **3**, which was isolated by preparative HPLC. [^19^F]-**3** was stable at physiological pH. Less than 1% defluoridation of [^19^F]-**3** was observed following 7 days of room temperature incubation with fetal bovine serum. The detailed synthesis is described in supporting information.

### Cell culture

The murine gliosarcoma cell line (9 L/lacZ), the murine endothelioma cell line (bEnd.3), and the human microvascular endothelial cell line (HMEC-1) were purchased from ATCC and cultured in DMEM medium, supplemented with 10% (FBS, Seradigm, USA) and 100 U/mL penicillin/streptomycin (Gibco, USA), at 37 °C in a humidified incubator. Cell cytotoxicity assessment methodology is described in supporting information.

### A note on IR783-AMBF_3_ nomenclature due to the use of a PET-active multimodality imaging probe

In this manuscript, the notation “[^18^F]-” on “[^18^F]-IR783-AMBF_3_” indicates that a radioactive mixture of fluoride-18 (<1%) and fluoride-19 (>99%) containing IR783-AMBF_3_ is used. [^18^F]-IR783-AMBF_3_ is visible on both fluorescent devices and PET/CT or PET/MRI devices. [^18^F]-IR783-AMBF_3_ and IR783-AMBF_3_ are electronically identical. The two molecules differ by only one neutron and the mixture is radioactive. The notation, IR783-AMBF_3_, without the “[^18^F]-”, denotes a formulation containing only fluorine-19 atoms. This formulation is not radioactive, non-PET active, but is fluorescent. Non-radioactive IR783-AMBF_3_ (as indicated by the lack of “[^18^F]”) is safer to handle and can be manipulated in facilities without radiation hazard protection standards.

### In vitro fluorescent imaging

Red blood cells (RBCs) isolation was performed as described previously [23]. RBCs were collected from a Balb/c mouse and suspended in sterilized PBS-containing heparin (Product#27602, Fresenius Kabi USA). Solutions of 1.5 × 10^9^ RBCs were mixed with different concentrations of IR783-AMBF_3_ (6.25–50 μM) or 25 μM of IR783-AMBF_3_ and mixed with different RBC counts (1.5 × 10^6^ to 1.5 × 10^9^) in an Eppendorf tube. Fluorescent images were measured using an In-Vivo imager (Bruker, Billerica, MA, USA) at excitation/emission acquisition settings of 760/830 nm. Fc-AMBF_3_ at the similar concentrations was used as a control. The excitation/emission acquisition settings for Fc-AMBF_3_ was 450/535 nm.

### CSF-rhinorrhea fluorescent imaging

Male Sprague-Dawley rats (6–7 weeks, 200–250 g) were purchased from Charles River Laboratories (Wilmington, MA, USA). Intrathecal catheterization was performed on rats by inserting a catheter (PE10 (0.26 mm I.D., 0.60 mm O.D), Instech Laboratories Inc., Plymouth Meeting, PA) into the subarachnoid space of spinal cord between L5-L6, as described previously [20]. A 22-G needle was passed through the left nostril to pierce the cribriform plate to induce CSF leakage from the olfactory bulb into the paranasal sinus in spaces that also contained blood. Following CSF leak creation, 10 μM IR783-AMBF_3_ (300 μL, 2.9 μg) was intrathecally injected into the rat, and CSF-rhinorrhea was collected on a gauze for fluorescent imaging. In control experiments, equimolar quantities of Fc-AMBF_3_ were used as a control.

### PET/MRI Scanning

A custom-built MRI compatible PET scanner with high-gain Silicon photomultiplier (SiPM) array detectors and clinical PET processing electronics (Cardinal, Siemens Molecular Imaging, Knoxville, TN) was used for the PET/MRI scan. This second generation PET/MRI scanner is considerably more thermal stable and less MRI sensitive than the first generation scanner [24]. The rat was anesthetized by 2% isoflurane and its respiration was monitored by a physiological monitoring system (SA Instruments, Stony Brook, NY). An intrathecal catheter line was placed before the animal was placed in the PET scanner. Two minutes after the start of PET data acquisition, 300 μL of 100 μCi [^18^F]-IR783-AMBF_3_ (2.9 μg) was infused into the catheter with an auto-injector at 10 μL/min for 30 min. ^18^F-PET data was continuously acquired for the same duration. After the PET scan, the animal was transported in the same cradle, in the same position, to the adjacent MRI scanner under anesthesia. Rat head and upper body MRI was performed on the 7-T MRI scanner using a rat brain coil for detection and a volume coil for RF excitation (Bruker Biospin Corp., Billerica, MA). After scout images were taken, 2D sagittal and coronal T2-weighted rapid acquisition with relaxation enhancement (RARE) images of the head and upper body were acquired. The FOV was 8 × 5 cm with a data matrix of 384 × 256, 20 1-mm slices, TR is 2.2 s and TE 50 ms, 8 averages, and a total acquisition of 10 min. PET data processing was achieved with home-written ordered subset expectation-maximization (OSEM) algorithm using the filtered back projection reconstruction method [25].

### PET/CT Imaging

PET/CT scanning was performed on a Siemens Inveon PET/CT scanner (Siemens, Malvern, PA, USA). The rats (*n* = 3), bearing an intrathecal catheter (PE10), were anesthetized with 2.0–2.5% isoflurane. Then a 10 min CT/2 h PET scan was obtained, and 300 μL of 100 μCi [^18^F]-IR783-AMBF_3_ was injected through catheter within 1 min during the PET phase. After scanning, the rats were sacrificed with CO_2_ overdose followed by bilateral thoracotomy. PET/CT was processed with Amide v1.0.4 and Inveon Research Workplace.

### Statistical analysis

Values were shown as mean ± SD for all experiments. Unpaired 2-tailed Student’s *t* test was performed to assess statistical significance of the results and *P* values less than 0.05 were considered significant.

## Results

### Synthesis and Characterization of IR783-AMBF_3_

We incorporated an alkylammoniomethyltrifluoroborate trap (AMBF_3_) onto IR-783 [26, 27] to give an IR-783 derivative, IR783-AMBF_3_. The synthesis of IR783-AMBF_3_ is described in Scheme 1, and the characterizations of IR783-AMBF_3_ by ^1^H NMR, ^13^C NMR, ^19^F NMR, UPLC-MS, and HRMS are reported in supporting information. A mass of 11 mg was obtained starting with 100 mg of IR-783 in a 3-step chemical synthesis. The AMBF_3_ and its linker did not alter IR-783’s fluorescent properties, as the optical properties of IR783-AMBF_3_ were similar to IR-783 (pH 7.4, 1 × PBS, Supporting Table 1). At physiological pH (1 × PBS), IR783-AMBF_3_ had an extinction coefficient of 196,000 M^−1^ cm^−1^ (εmax = 779 nm) and an excitation maximum *λ* = 778 nm, with Stokes shift of 15 nm, while IR-783 had an excitation maximum *λ* = 780 nm, with Stokes shift of 22 nm (Supporting Table 1). IR783-AMBF_3_ had a quantum yield of *φ* = 0.186, similar to that of IR-783 (*φ* = 0.183) (Supporting Table 1). The detailed fluorescence spectrum of IR783-AMBF_3_ and IR-783 are shown in the supporting information.

### Radiochemistry

The radiolabeling of IR783-AMBF_3_ (**3**, Scheme 1) was performed in 30 min. In a typical synthesis, 9.2 mCi of [^18^F]-IR783-AMBF_3_ (**4**) (molar activity of 185 mCi/μmol, RCY = 23%, decay uncorrected) was obtained starting with 40.5 mCi of [^18^F]-fluoride ion (Scheme 1, d). Isotope exchange was undertaken in one step under aqueous, acidic pH conditions (pH = 2.0, pyridazine−HCl buffer, 10 μL) [28] and proceeded quickly (10–15 min) at high temperatures (90–100 °C). Unreacted [^18^F]-fluoride ion was removed by passing the [^18^F]-IR783-AMBF_3_ (**4**) reaction mixture through a prewashed (2 mL ethanol followed by 5 mL deionized water) C18 cartridge (Waters no. 186005125). Unreacted [^18^F]-fluoride ion was flushed from the cartridge with 20 mL water. [^18^F]-IR783-AMBF_3_, bound to the cartridge, was eluted with a 3.0 mM HCl in ethanol (300 L, 99%). All steps involving the cartridge were performed using a syringe pump set to deliver at a 40 mL/h flow rate. Resulting [^18^F]-IR783-AMBF_3_ in acidic ethanol was immediately diluted 10- to 20-fold with 1 mM 1 × PBS and filtered through a 0.22 μm filter. The reverse-phase HPLC of the resulting pH 7.4 filtrate showed high purity of [^18^F]-IR783-AMBF_3_. The resulting filtrate was injected intrathecally.

### PET and fluorescent visualization of IR783-AMBF_3_ in the CSF-containing cisterns

**Fig. 1:**
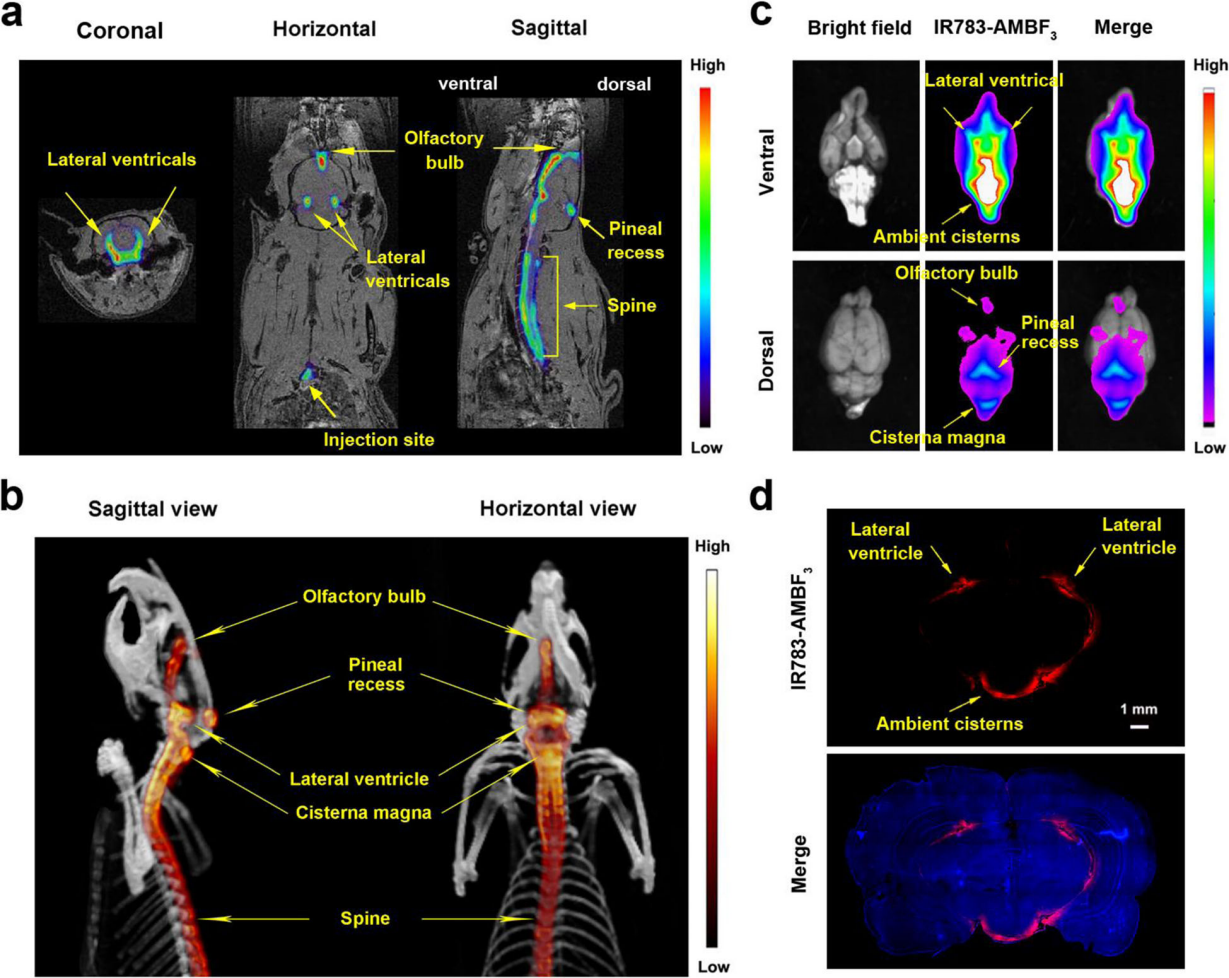
PET/MRI, PET/CT, and fluorescent visualization of IR783-AMBF_3_ distribution in the CNS after intrathecal injection. **a** A PET/MR scan of a rat following intrathecal introduction of [^18^F]-IR783-AMBF_3_ shows contrast that is clearly visible in the spine, pineal recess, pituitary recess, and olfactory bulb. **b** Maximum intensity projection PET/CT image of intrathecally injected [^18^F]-IR783-AMBF_3_ at 5 min post injection. IR783-AMBF_3_ distributes to the CSF-containing space similarly in **a** PET/MRI and **b** PET/CT. **c** After PET/CT imaging, the rat brain was collected for fluorescent imaging. **d** Ex vivo fluorescent histology of a coronal section of the brain in a healthy rat that has been injected with IR783-AMBF_3_ (10 μM) after 20 min. DAPI (blue) and IR783-AMBF_3_ fluorescent signal (red) show IR783-AMBF_3_ in the ventricles where CSF is normally present at quantity

To determine [^18^F]-IR783-AMBF_3_ utility in imaging CSF flow, we first evaluated [^18^F]-IR783-AMBF_3_ contrast distribution in the CNS following intrathecal administration (between the L5-L6 vertebrae) on PET and fluorescent imaging devices. Following L5-L6 intrathecal introduction, [^18^F]-IR783-AMBF_3_ presence was observed in the ambient cisterns, pineal recess, pituitary recess, and olfactory bulb both by PET/MRI (Fig. 1a) and PET/CT (Fig. 1b) imaging, which distributed the same way as a fluorescein-conjugated AMBF_3_ (Fc-AMBF_3_, Scheme 1) [20, 21]. A dynamic video of intrathecal injection of [^18^F]-IR783-AMBF_3_ PET/MRI is included in Supporting Video 1. It should be noted that there was no contaminating free [^18^F]-fluoride ion after radiolabeling or before injection. Defluoridation of IR783-AMBF_3_ was not observed. [^18^F]-fluoride ion accumulation at the bone was not observed by PET/MRI or PET/CT. This lack of bone uptake demonstrates reliable synthesis, IR783-AMBF_3_ radiolabeling, and molecular stability after intrathecal injection. Following PET/CT scanning, rats were sacrificed by CO_2_ overdose and rat brains were collected and analyzed by ex vivo fluorescent imaging and high-magnification fluorescent histological analysis (Fig. 1c). Fluorescent signal was visible in the basal ambient cisterns, which was consistent with the PET images. Notably, two lateral ventricles were observable through the unprocessed brain in the IR783-AMBF_3_ fluorescent channel. In our previous publication using Fc-AMBF_3_ [20, 21], these ventricles were difficult to visualize without sectioning the brain due to the strong absorption of exciting and emitted short wavelength photons by overlying brain tissue [2]. Fluorescent histology of a coronal section (− 19.11 mm, ant. ac) showed IR783-AMBF_3_ in the ventricles where CSF is normally present (Fig. 1d). These data imply that newly designed IR783-AMBF_3_ can serve as a multimodal contrast agent for delineating CSF-containing space by both PET and fluorescent imaging.

### IR783-AMBF_3_ was superior to Fc-AMBF_3_ in diagnosing CSF leak when hemorrhage was present

**Fig. 2:**
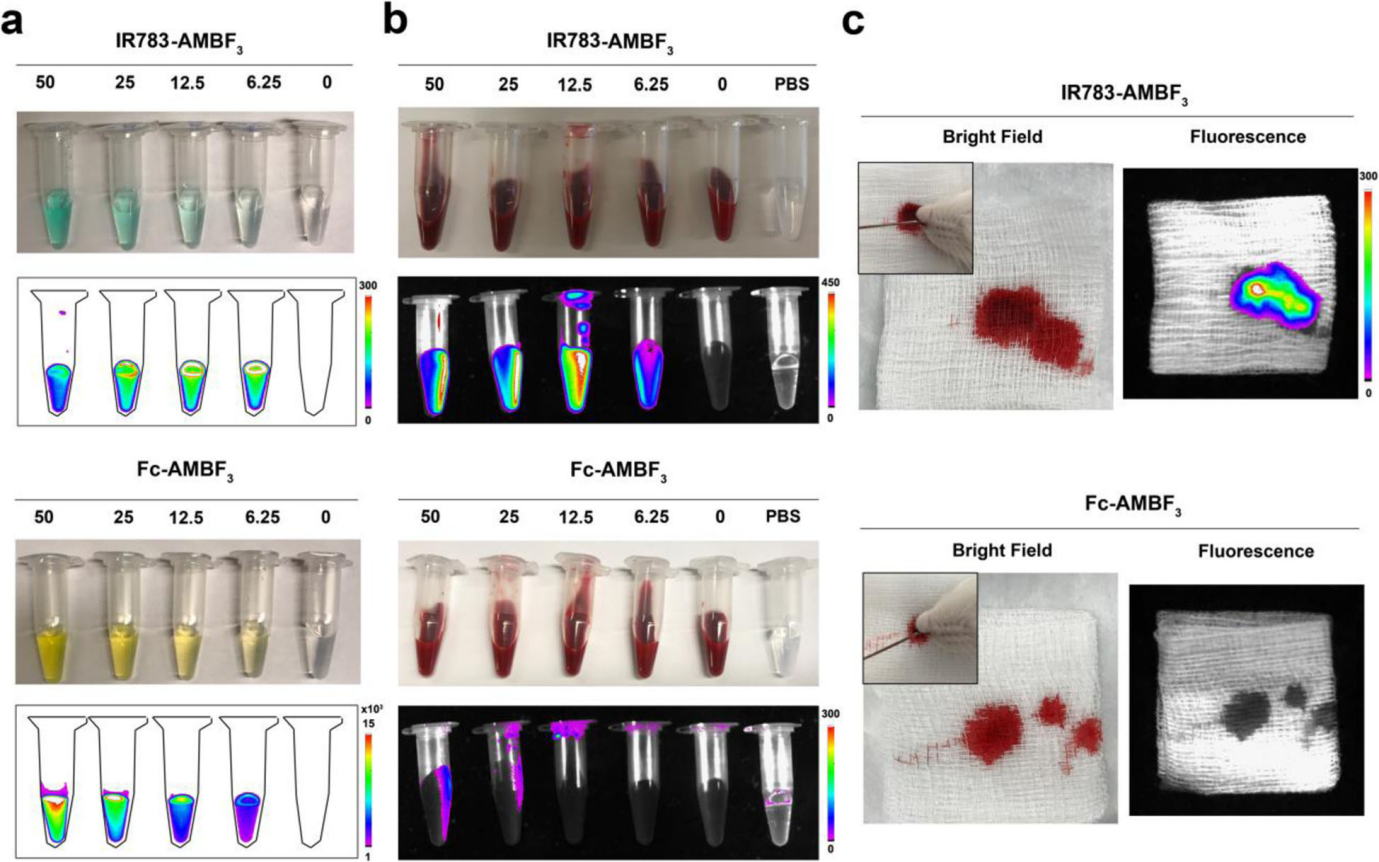
RBC-mediated quenching of Fc-AMBF_3_ but not IR783-AMBF_3_ fluorescence. Fluorescence imaging of different concentrations of IR783-AMBF_3_ or Fc-AMBF_3_ in PBS, **a**, or when mixed with 1.5x10^9^ RBC/per tube, **b**. When mixed with blood, Fc-AMBF_3_ fluorescence is not visible, while IR783-AMBF_3_ fluorescence is clearly visible. Fluorescence imaging of blood contaminated CSF-containing rhinorrhea following the creation of a CSF leak using a 22G needle **c**. Excitation/emission filters for Fc-AMBF_3_ and IR783-AMBF_3_ were set at 450/535 nm and 760/830 nm, respectively. Note that in a CSF leak where RBC is not present, Fc-AMBF_3_ is visible by fluorescence [20]

In a prior study, we reported that Fc-AMBF_3_, a fluorescein PET/Fluorescent agent, is useful for visualizing a paranasal-sinus CSF leak in rats by PET and fluorescence [20]. We concluded that Fc-AMBF_3_ (excitation/emission = 495/511 nm) can be used to guide surgical CSF leak repair in endoscopic procedures in real time or to inform on the qualitative status of a CSF tear [29, 30]. However, CSF-bearing rhinorrhea collected from rats with a CSF leak was only fluorescently visible when the CSF was not contaminated with blood. A drawback to Fc-AMBF_3_ is that the absorption of red blood cells (RBCs) can quench Fc-AMBF_3_ fluorescence significantly (Fig. 2a, b, bottom) (Fig. S1b). Fortunately, IR783-AMBF_3_ fluorescence was not quenched by RBCs (Fig. 2a, b, top), even when mixed with an increasing number of RBCs (Fig. S1a). To mimic the potential clinical ability of IR783-AMBF_3_ in confirming CSF leak, in the presence of contaminating blood, an anterior skull base CSF leak in a rat with significant accompanying hemorrhage was created. A 22-G needle was inserted into left nostril of the deceased rats to induce the CSF leakage. As shown in Fig. 2c, CSF that was present in rhinorrhea collected from nostrils and month of rats bearing intrathecal IR783-AMBF_3_ could not be visualized by the naked eye when it was mixed with blood. However, the CSF leak was able to be imaged and identified by IR783-AMBF_3_ fluorescence even in the absence of massive bleeding (top). On the contrary, Fc-AMBF_3_ fluorescence was not visible in a rat rhinorrhea containing significant quantities of blood (bottom). The above data demonstrate that IR783-AMBF_3_ can be used in the identification of CSF leak in particularly traumatic situations: e.g. to assist surgeons in the rapid, accurate diagnosis and treatment of CSF lesions in the emergency setting.

### IR783-AMBF_3_ fluorescence was visible through skin in a rat bearing a lumboperitoneal shunt

**Fig. 3:**
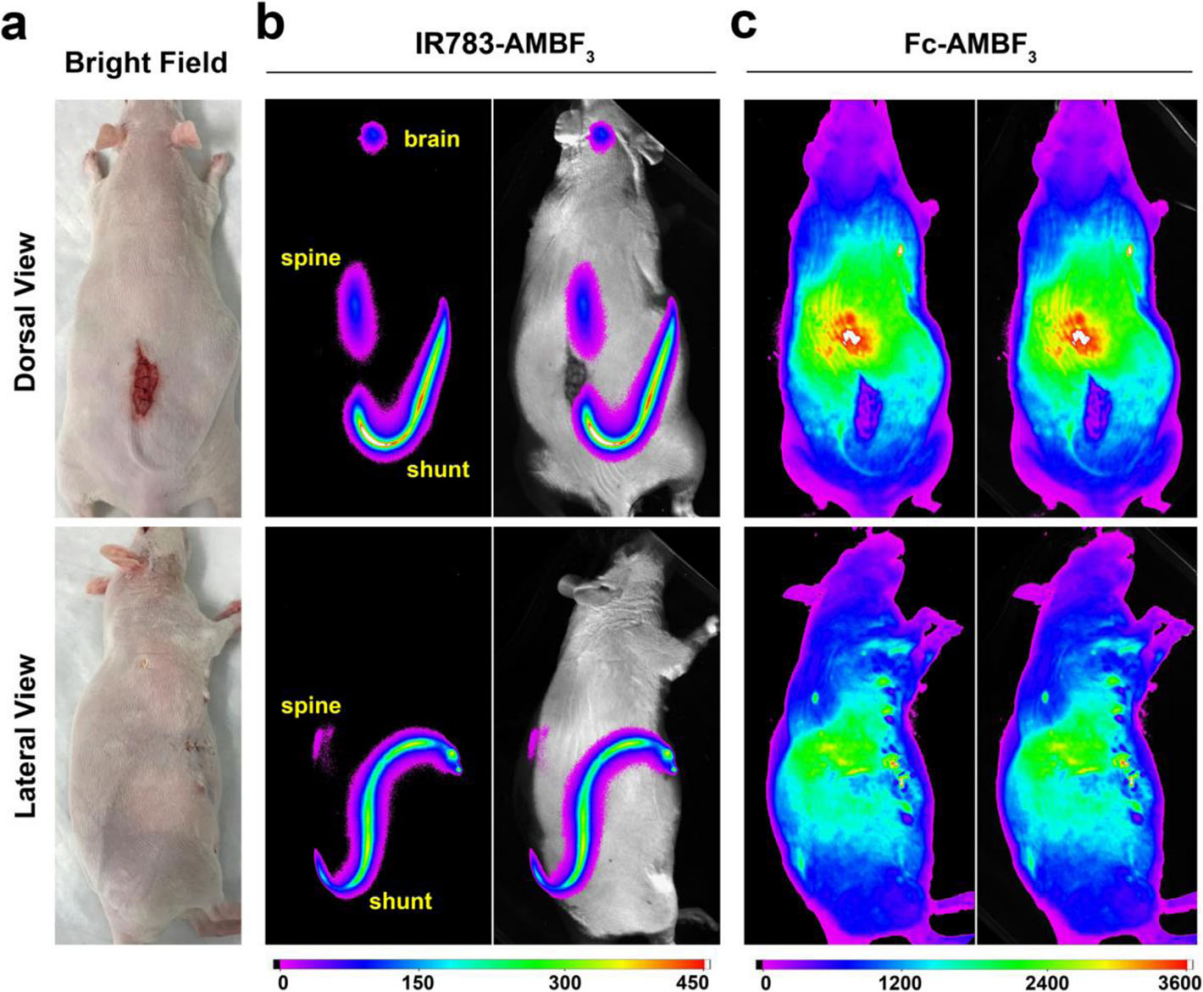
Non-radioactive IR783-AMBF_3_ can be visualized through deep tissue in the fluorescence mode. **a** Bright field images of rats (*n* = 2) with LP shunts that run superficially, under the skin of a shaved rat. **b** In vivo fluorescent images of the shunt in a rat after filling the shunt with IR783-AMBF_3_ (10 μM). **c** (Control) Comparative in vivo fluorescent imaging of a shunt filled with Fc-AMBF_3_ placed in a rat (1 mM, L5-L6). IR783-AMBF_3_ fluorescence is visible in the shunt, spine, and brain, and through skin, bone, and a viable shunt

To investigate IR783-AMBF_3_ utility in observing CSF flow through a lumboperitoneal (LP) shunt, an LP shunt was placed in rats [21]. One end of the catheter (PE60) was inserted into the CSF space between L5 and L6 of a rat, and the outlet of this catheter was run subcutaneously along the abdomen, before being directed in the abdominal cavity (Fig. 3a). IR783-AMBF_3_ was injected through the cisterna magna [21] and its flow was visualized in the fluorescent mode. IR783-AMBF_3_ fluorescence was observable in vivo, through the skin (and some bone) and in the shunt, spine, and brain, demonstrating that IR783-AMBF_3_ correctly distributed in the CNS via normal CSF flow (Fig. 3b). In control experiments performed with Fc-AMBF_3_, no significant fluorescent signal in the shunt, spine, or brain could be observed through the skin (Fig. 3c). Most importantly, IR783-AMBF_3_ clearance from CNS into the peritoneal cavity could be monitored through the skin by fluorescence imaging (Fig. S2). The fluorescent intensity of IR783-AMBF_3_ in the shunt decreased over time. The fluorescent intensity in the brain and peritoneal cavity first increased as IR783-AMBF_3_ diffused into these structures with CSF flow, and then decreased as IR783-AMBF_3_ cleared from the brain of rats (Fig. S3). This model is relevant in the clinical setting as ventriculoperitoneal shunts are run in patients, subdurally, from ventricles in the brain to the peritoneum. It was noted that the fluorescence signal in the kidney also increased after intrathecal administration of IR783-AMBF_3_ and then decreased over time (Fig. S3). These fluorescence imaging data suggest that intrathecally injected [^18^F]-IR783-AMBF_3_ clears from the CSF.

### IR783-AMBF_3_ was safe

**Fig. 4:**
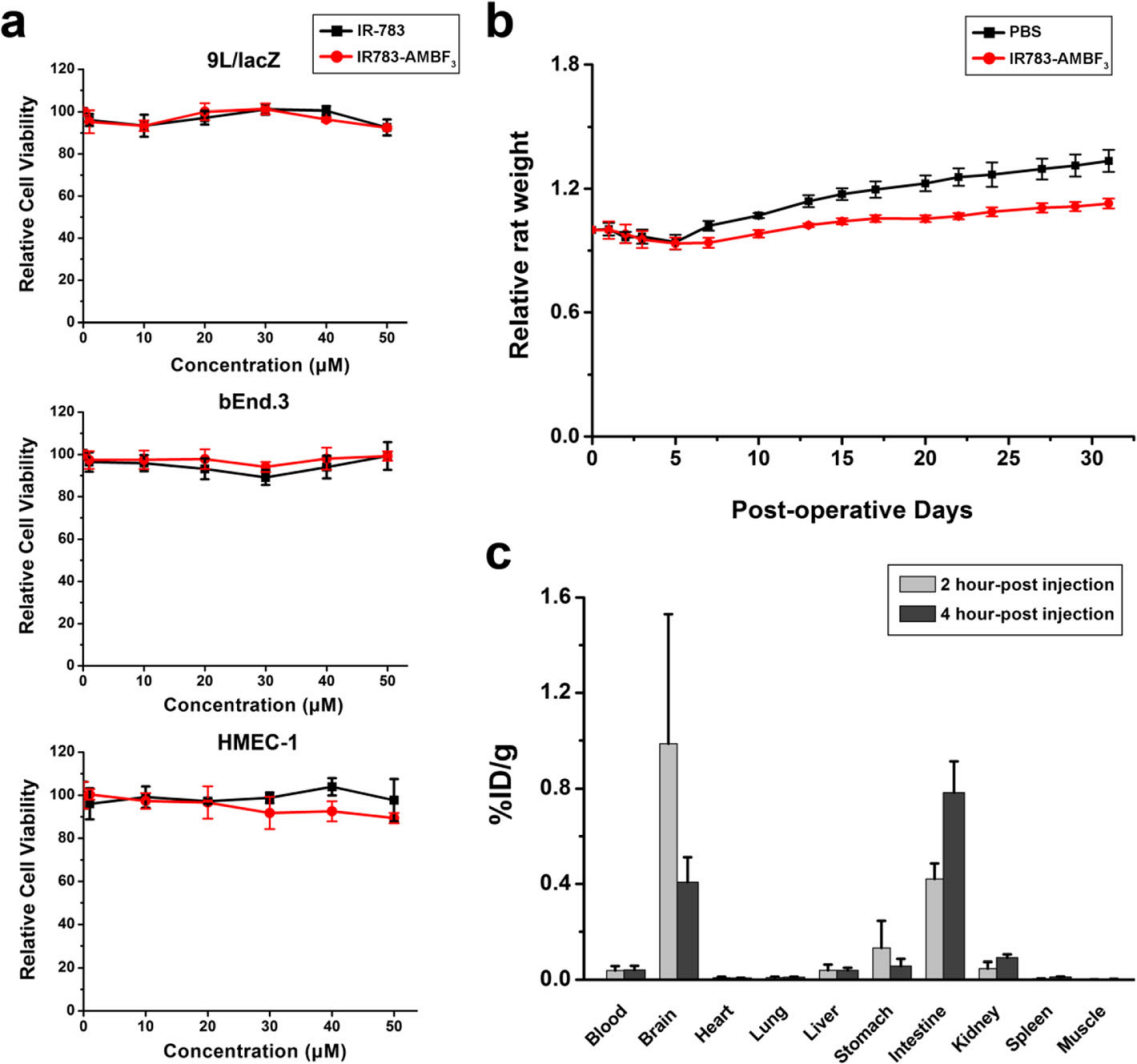
Safety studies of IR783-AMBF_3_. **a** Effect of IR783-AMBF_3_ on murine gliosarcoma (9 L/lacZ), murine endothelioma (bEnd.3) and human microvascular endothelial (HMEC-1) cell viability. Cells were treated with different concentrations of IR-783 (black line) or IR783-AMBF_3_ (red line) for 24 h. **b** Relative mean weight of rats after intrathecal injection of IR783-AMBF_3_ at 150 μM (*n* = 4). Error bars are ± SD. **c** Biodistribution data collected in different tissues shows that intrathecally injected IR783-AMBF_3_ clears from the CSF and blood quickly and is ultimately excreted and through the renal and hepatic systems (*n* = 3). Error bars are ± SEM

The safety profile of IR783-AMBF_3_ was evaluated in different cell lines and in healthy rats. IR783-AMBF_3_ did not affect proliferation at concentrations as high as 50 μM following the incubation of IR783-AMBF_3_ with cells for 24 h, suggesting that IR783-AMBF_3_ does not induce cytotoxicity at concentrations under 50 μM (Fig. 4a). To evaluate the intrathecal toxicity of IR783-AMBF_3_, rats (*n* = 4) were intrathecally injected with 45 nmol (15-fold higher than the imaging dose), and rat weight was monitored every other day for up to 1 month; normal recovery following intrathecal catheterization surgery (Fig. 4b) and post-surgical weight gain were observed in the rats. To confirm that IR783-AMBF_3_ clears entirely from the CSF, rats were injected intrathecally with IR783-AMBF_3_ and their organs were collected at 2 h and 4 h post-injection (*n* = 3) for γ-scintillated biodistribution data collection (Fig. 4c). Biodistribution data showed that IR783-AMBF_3_ was cleared from the brain and spine, into the intestines and kidneys, suggesting that all intrathecally injected IR783-AMBF_3_ was cleared from the CNS, and excreted through hepatic and renal routes.

## Discussion

The direct radiochemical introduction of an ^18^F atom onto a cyanine dye using traditional carbon fluoride bond formation strategies requires non-trivial or multistep approaches. These requirements demand automated synthesis, greater quantities of starting radioactivity, and lower chemical and radiochemical yields. Heptamethine cyanines are particularly unstable in basic conditions. We describe a convenient synthesis of a PET-emitting heptamethine cyanine using aqueous, acidic ^18^F-isotopic exchange radiolabeling of IR783-AMBF_3_ [31, 32]_._ IR-783 that is modified to bear a 4-carboxy phenylboronic acid provides a handle for further modification via Suzuki coupling (C–C bond formation). Isotopic exchange acidic radiolabeling gave the high-yield transfer of [^18^F]-fluoride ion onto [^18^F]-IR783-AMBF_3_. The resulting radiolabeled, near-infrared wavelength fluorescent, dye-mixture contains [^18^F]-IR783-AMBF_3_ for visualization by PET (both PET/CT and PET/MRI) and non-radioactive, non-PET visible IR783-AMBF_3_ for fluorescence imaging (Fig. 1).

### Rational design of IR783-AMBF_3_

The use of AMBF_3_ (dimethylammonium methyltrifluoroborate) ^18^F-isotopic exchange technology allows us to employ a stable, fluoride-19 containing, IR783-AMBF_3_ radiochemical precursor to synthesize radioactive [^18^F]-IR783-AMBF_3_ [32, 33]. Because of the AMBF_3_ isotopic exchange technology employed, IR783-AMBF_3_ is also the stable radiochemical precursor to [^18^F]-IR783-AMBF_3_. [^18^F]-IR783-AMBF_3_ and IR783-AMBF_3_ are electronically identical. i.e. No biological system can accurately distinguish between a molecule of [^18^F]-IR783-AMBF_3_ and IR783-AMBF_3_. This means that non-radioactive, fluoride-19 containing, IR783-AMBF_3_ can substitute for [^18^F]-IR783-AMBF_3_ in fluorescent-only studies or in toxicology studies (but not dosimetry studies). Additionally, IR783-AMBF_3_ is hydrophilic and is low in molecular weight (MW < 1000). This hydrophilicity, conveyed by the zwitterionic AMBF_3_, will prevent IR783-AMBF_3_ from adhering to the meninges lining the CSF pathways, thus ensuring that IR783-AMBF_3_ is cleared from the CSF and the body after a reasonable time, post-scan. Finally, IR783-AMBF_3_ has a low molecular weight that resembles the molecular weights of both indocyanine green (ICG) and fluorescein, two current FDA approved agents for visualizing vascular flow in the fluorescent mode. In this study, when injected into the CSF, IR783-AMBF_3_ allowed the CSF to be imaged in a rat model of CSF leak (Fig. 2) as well as allowed CSF flow to be imaged through a patent lumboperitoneal shunt (Fig. 3). In vitro or in vivo, IR783-AMBF_3_ did not show any toxicity and was rapidly (< 2 h) cleared from the blood (Fig. 4).

### Potential applications of IR783-AMBF_3_

IR783-AMBF_3_ is designed to roughly resemble the FDA approved agent indocyanine green (ICG), which is used in angiography and lymphangiography by ophthalmologists, interventional radiologists, and surgeons (sentinel node removal). IR783-AMBF_3_ shares a molecular weight and a hydrophobicity that is similar to ICG. These properties were incorporated into the design of IR783-AMBF_3_, in the hopes that IR783-AMBF_3_ could serve as a substitute to ICG in future studies. For example, IR783-AMBF_3_ potentially could allow for guided surgery on FDA-approved intraoperative fluorescent robotic devices [22], and to assist in the non-invasive through-skin evaluation of devices placed in the CNS [34]. In addition to being visible on the same fluorescent devices used to visualize ICG, IR783-AMBF_3_ is also useful in PET scanning and will allow the molecularly-coherent-visualization of flow on PET/CT, PET/MRI and fluorescent devices. In CSF applications, drug and IR783-AMBF_3_ co-delivery could be used to know exact CSF flow rates to allow for more accurate drug delivery in treating leptomeningeal disease [21], or to precisely localize injury sustained by the central nervous system (CSF leak).

### Advantages of IR783-AMBF_3_ vs. other modalities

CSF leaks can have a variety of appearances depending on their cause and rate leakage. Leak localization, characterization, and mitigation are very important to aid both diagnosis and treatment. As a result, a number of imaging tests have been used to detect these leaks, each with its own strengths and weaknesses [35]. CT myelography and cisternography are the commonly used modalities in diagnosing CSF leaks [36]. MR cisternography has the advantage of being non-invasive and of not involving any ionizing radiation [35]. However, MR cisternography typically does not localize the exact leak site and suffers from artifacts. In addition, it can be difficult to distinguish CSF from sinusitis [37]. MR contrast myelography is superior to non-contrast MR; however, the introduction of high-dose gadolinium into the intrathecal space may bring up potential neurotoxicity in the CNS [18, 38, 39] and the long-term safety of intrathecal gadolinium is not conclusively known. Intrathecal gadolinium is not approved by the U.S. FDA currently. Another alternative approach for diagnosing CSF leak is ^111^In-diethylene triamine pentaacetic acid (DTPA) intrathecal injection followed by single photon emission computed tomography (SPECT) imaging [40, 41]. The advantage of ^111^In-SPECT is its prolonged monitoring capability—up to or greater than 72 h for the diagnosis of intermittent CSF leak [40]. However, this exam gives relatively low spatial resolution and sensitivity. On the contrary, IR783-AMBF_3_ PET/MR is potentially a more sensitive and quantitative means of imaging CSF leak, since PET can produce higher resolution images than its ^131^I, ^111^In or ^99m^Tc SPECT contrast counterparts [42]. Low-mass injections (< 1 mg) of IR783-AMBF_3_ PET/CT are unlikely to have the same issues associated with the introduction of intrathecal gadolinium MR contrast in the CNS. IR783-AMBF_3_ is superior to standalone PET due to its near-infrared fluorescence properties. IR783-AMBF_3_ can immediately identify the precise anatomical location of CSF tears in patients with severe brain trauma in the emergency room using FDA-approved fluorescent imaging systems. There is no current universal approach for imaging CSF leaks most efficiently, and a single, superior imaging modality currently does not exist [1].

### Advantages of near-infrared fluorescent IR783-AMBF_3_ vs. fluorescent Fc-AMBF_3_

We previously established the use of Fc-AMBF_3_ to image CSF flow [21]. Like Fc-AMBF_3_, IR783-AMBF_3_ can be used to resolve CSF flow deep within the CNS in the PET mode. In this study, we observed three significant advantages of IR783-AMBF_3_ over Fc-AMBF_3_: (1) IR783-AMBF_3_ can be observed in shunts, through the skin, and in subdural-run lumboperitoneal shunts, whereas Fc-AMBF_3_ fluorescence cannot be observed through the skin of rats. Fc-AMBF_3_ fluorescence is only visible in superficially run LP shunts (Fig. 3, Fig. S3). It is impractical to run a LP shunt superficially in a patient, as LP shunts that are superficially run in patients introduce a significant likelihood of infection and shunt dislocation. (2) IR783-AMBF_3_ fluorescence is visible when mixed with blood, while Fc-AMBF_3_ fluorescence is not visible in the presence of blood (Fig. 2). In scheduled surgeries, the presence of blood is highly regulated and is therefore generally not an issue. However, IR783-AMBF_3_ would be superior in an emergency room setting where a significant presence of blood can obscure trauma involving the CSF. (3) The greatest advantage of IR783-AMBF_3_ is its current compatibility with currently FDA-approved intraoperative robotics which is already equipped to visualize IR783-AMBF_3_.

### Disadvantages of IR783-AMBF_3_ vs. Fc-AMBF_3_

IR783-AMBF_3_ (excitation/emission = 778/793 nm) requires more complicated instrumentation for its fluorescent observation. The wavelength of photon emitted by IR783-AMBF_3_ is not visible by the naked eye; therefore, a CCD/CMOS camera fitted with an optical surface is required to collect and report on the emitted, fluorescent photon. Additionally, a surgeon can only visualize IR783-AMBF_3_ on a digital screen. Unlike IR783-AMBF_3_, the fluorescent emission of Fc-AMBF_3_ (excitation/emission = 495/511 nm) is visible with inexpensive black-light illumination, by the naked eye, and without an emission filter; therefore, surgeons can observe Fc-AMBF_3_ directly in the operating field within a patient without costly equipment. Another drawback to IR783-AMBF_3_ is that the safety of IR783-AMBF_3_ in the CSF is unknown. Fc-AMBF_3_ is attractive for clinical trials as it is molecularly similar to fluorescein. Fluorescein is currently used and is known to be safe at < 50 mg doses for the purposes of CSF leak repair. The safety of IR783-AMBF_3_ is less understood; however, the noted advantages of IR783-AMBF_3_ may make its utility in CSF imaging more attractive vs. Fc-AMBF_3_.

## Conclusion

A new synthesis and ^18^F-radiolabeling of a dual PET/near-infrared fluorescence probe, IR783-AMBF_3_, is reported. Intrathecal injections of IR783-AMBF_3_ are tolerated in rats under 45 nmol dosage, and it can rapidly clear from the body within 2 h. IR783-AMBF_3_ can be used to non-invasively and accurately diagnose and monitor CSFL and CSF flow through lumboperitoneal shunts by both PET imaging and near-infrared fluorescence imaging. The ability to image CSF flow in deep tissue and in the presence of blood allows IR783-AMBF_3_ to serve as a powerful dual-modality agent for diagnosing both CSF-related diseases and imaging vascular flow.

## Supplementary information

**Additional file 1.** A dynamic [^18^F]-IR783-AMBF_3_ PET/MRI of a rat showing IR783-AMBF_3_-CSF flow into the brain (0–30 min) by a 300 μL, 10 μL/min [^18^F]-IR783-AMBF_3_ lumbar (L5–L6) infusion.

**Additional file 2.** Synthesis and chemical characterization of IR783-AMBF3, supporting materials, methods, and supporting figures.

## Data Availability

Please contact the authors for data or material requests.

## Abbreviations

9 L/lacZ: Murine gliosarcoma cell
ACN: Acetonitrile
AMBF3: alkylammoniomethyltrifluoroborate
bEnd.3: murine endothelioma cell
CNS: Central nervous system
CSF: Cerebrospinal fluid
CSFL: Cerebrospinal fluid leak
CT: Computed tomography
DMF: Dimethyl formamide
DMSO: Dimethyl sulfoxide
DTPA: Diethylene triamine pentaacetic acid
EDCI: 1-ethyl-3-(3-dimethylaminopropyl)carbodiimide hydrochloride
eq: Equivalents
EtOH: Ethanol
HCl: Hydrochloric acid
HMEC-1: human microvascular endothelial cell
HOBt: Hydroxybenzotriazole
HPLC: High performance liquid chromatography
ICG: Indocyanine green
ID: Inside diameter
KHF_2_: Potassium hydrogen fluoride
L5-L6: The space between fifth and sixth lumbar vertebrae
LP: Lumboperitoneal
MeOH: Methanol
MRI: Magnetic resonance imaging
MS: Mass spectrometer
NMR: Nuclear magnetic resonance
OD: Outside diameter
PBS: Phosphate-buffered saline,
PE: Polyethylene
PET: Positron emission tomography
RBCs: Red blood cells
SPECT: Single photon emission computed tomography
TFA: Trifluoroacetic acid
