# Supplementary material for: A near-infrared probe for non-invasively monitoring cerebrospinal fluid flow by 18F-positron emitting tomography and fluorescence
Source: EJNMMI Res. 2020 Apr 16;10:37. doi: 10.1186/s13550-020-0609-3 (PMC7163004; doi:10.1186/s13550-020-0609-3)
Supplement: Supplementary file 2 — Additional file 2. Synthesis and chemical characterization of IR783-AMBF3, supporting materials, methods, and supporting figures. [file 13550_2020_609_MOESM2_ESM.docx]

Supporting information

**A Near-infrared Probe for Non-invasively Monitoring Cerebrospinal Fluid Flow by ^18^F-Positron Emitting Tomography and Fluorescence**

Hua Guo^1,2,3#^, Harikrishna Kommidi^3#^, Carl H. Lekaye^4^, Jason Koutcher^4^, Martin S. Judenhofer^5^, Simon R. Cherry^5^, Amy P. Wu^6^, Oguz Akin^7^, Mark M. Souweidane^8^, Omer Aras^7^, Zhaohui Zhu^1,2*^ and Richard Ting^3*^

^1^ Department of Nuclear Medicine, Chinese Academy of Medical Sciences and Peking Union Medical College Hospital, Beijing, 100730, China.

^2^ Beijing Key Laboratory of Molecular Targeted Diagnosis and Therapy in Nuclear Medicine, Beijing 100730, China.

^3^ Department of Radiology, Molecular Imaging Innovations Institute (MI3), Weill Cornell Medical College, New York, NY 10065, USA.

^4^ Department of Medical Physics, Memorial Sloan Kettering Cancer Center, New York, NY, 10065, USA.

^5^ Department of Biomedical Engineering, University of California at Davis, Davis, CA, 95616, USA.

^6^ Department of Otolaryngology – Head & Neck Surgery, Northwell Health, Hofstra Northwell School of Medicine, New York, NY, 10075, USA.

^7^ Department of Radiology, Memorial Sloan Kettering Cancer Center, New York, NY, 10065, USA.

^8^ Department of Neurological Surgery, Weill Cornell Medicine, New York, NY 10065, USA.

^#^H.G. and H.K. contributed equally to this work

^*^Corresponding authors: Richard Ting, E-mail: [rct2001@med.cornell.edu](mailto:rct2001@med.cornell.edu) Zhaohui Zhu, E-mail: [13611093752@163.com](mailto:13611093752@163.com)

Table of contents

1. [^18^F]-IR783-AMBF_3_ synthesis scheme 3

2. Synthesis of (1), (2), and (3) 4

3. Proof of synthesis 6

3.1 Characterization of (1) 6

3.1.1 ^1^H NMR (DMSO-d_6_, 500 MHz, 21 °C) 6

3.1.2 ^13^C NMR (DMSO-d_6_, 125 MHz, 21 °C) 7

3.1.3 HRMS [M]^+^ 8

3.2 Characterization of (2) 9

3.2.1 ^1^H NMR (DMSO-d_6_, 500 MHz, 21 °C) 9

3.2.2 ^13^C NMR (DMSO-d_6_, 125 MHz, 21 °C) 10

3.2.3 HRMS [M+H]^+^ 11

3.3 Characterization of (3) 12

3.3.1 ^1^H NMR (DMSO-d_6_, 500 MHz, 21 °C) 12

3.3.2 ^13^C NMR (DMSO-d_6_, 125 MHz, 21 °C) 13

3.3.3 ^19^F NMR (DMSO-d_6_, 470 MHz, 21 °C) 14

3.3.4 UPLC-MS Chromatogram 15

3.3.5 HRMS [M+H]^+^, [M-F]^+^ 16

4. Fluorescence properties of (IR-783, IR783-AMBF_3_) 17

4.1 Fluorescence spectrum of IR-783 in absolute ethanol 17

4.2 Fluorescence spectrum of (IR783-AMBF_3_) in absolute ethanol 18

4.3 Fluorescence spectrum of IR-783 in 1x PBS, pH 7.4 19

4.4 Fluorescence spectrum of (IR783-AMBF_3_) in 1 x PBS, pH 7.4 20

4.5 Reverse-phase HPLC of radiolabeled [^18^F]-IR783-AMBF_3_ 21

5. Supporting Table 1. Optical properties of IR783-AMBF_3_ were identified along-side IR-783 in pH=7.4 (1x PBS) and in absolute ethanol 22

6. Supporting materials and methods 23

6.1 Cytotoxicity assessment 23

6.2 Tissue distribution 23

6.3 *In vivo* toxicity assessment 23

6.4 Histological analysis 23

6.5 Lumboperitoneal shunt 24

7. Supporting figures 25

**1. [^18^F]-IR783-AMBF_3_ synthesis scheme**

**2. Synthesis of (1), (2) and (3)**

***2-((E)-2-((E)-4'-carboxy-6-(2-((E)-3,3-dimethyl-1-(4-sulfobutyl)indolin-2-ylidene)ethylidene)-3,4,5,6-tetrahydro-[1,1'-biphenyl]-2-yl)vinyl)-3,3-dimethyl-1-(4-sulfobutyl)-3H-indol-1-ium (1):*** A solution of IR-783 (100 mg, 133 µmol) in water/DMF (10:1) (2 mL) was degassed through an argon purge. 4-carboxyphenylboric acid (266 µmol) followed by Pd(PPh_3_)_4_ (2.6 µmol) was added and the reaction mixture was heated to 100 ºC for 10 h or until complete consumption of IR-783 starting material was observed by HPLC. Reaction progress was monitored by UPLC/MS. The reaction mixture was allowed to cool to room temperature, and water was removed under vacuum. The heptamethine cyanine-acid **1** (60 mg) was isolated as a pure solid following precipitation with MeOH/acetone, washing with acetone (2 x 5 mL), and crystallization from EtOH. ^1^H NMR (500 MHz, DMSO-d6): δ 8.35 (t, 1H, J = 8.0 Hz), 7.48 (d, 2H, J = 7.0 Hz), 7.43 (d, 2H, J = 8.0 Hz), 7.40-7.31 (m, 4H), 7.14 (t, 4H), 7.01 (d, 2H, J = 14.0 Hz), 6.26 (d, 2H, J = 9.0 Hz), 4.12 (m, 5H), 2.71(bs, 1H, J = 7.5 Hz), 2.48-2.45 (m, 4H), 1.96 (bs, 2H), 1.76-1.64 (m, 8H), 1.09(s, 12H); ^13^C NMR (125 MHz, DMSO-d6): 171.1, 167.0, 159.7, 146.7, 143.7, 142.0, 140.6, 130.5, 130.2, 129.6, 129.4, 128.4, 124.5, 122.4, 111.1, 100.3, 50.7, 48.1, 43.4, 26.9, 22.5, 20.8; HRMS (ESI)^-^: m/z calculated for [M]^+^: C_45_H_53_N_2_O_8_S_2_^+^: 813.3243; found: 813.3761

***2-((E)-2-((E)-6-(2-((E)-3,3-dimethyl-1-(4-sulfobutyl)indolin-2-ylidene)ethylidene)-4'-((2-(dime-thylamino)ethyl)carbamoyl)-3,4,5,6-tetrahydro-[1,1'-biphenyl]-2-yl)vinyl)-3,3-dimethyl-1-(4-sulfobutyl)-3H-indol-1-ium (2):*** To a magnetically stirred solution of acid **1** (50 mg, 61 µmol) in 2 mL of dry DMF in an oven-dried 10 mL round bottom flask, N,N-dimethyl ethyl amine (11 mg, 123 µmol), 6 µL of pyridine, and 1-hydroxybenzotriazole (13 mg, 92 µmol) was added before amide bond formation was initiated by adding EDCI (36 mg, 185 µmol). The reaction was allowed to stir for 5 h at 27 °C. The formation of IR783-amide, **2**, was observed by UPLC/MS. The resulting solution was diluted with acetone (20 mL), to give a precipitate that was isolated through centrifugation. The isolated precipitate was washed with 5% ethanol in acetone (5 mL x 5) to give the desired IR783-amide **2** (38 mg, 69%, purity 90%). The compound **2** (20 mg) was purified by preparative HPLC using a H_2_O/ACN (0.05% TFA), 40 min elution gradient at a flow rate of 12 mL/min. A linear gradient of increasing ACN from 10% to 70% between 0 and 30 min, followed by a linear increase of ACN from 70% to 90% between 30 and 40 min, was used to elute **2**. Fractions containing 2 were lyophilized in vacuo to yield pure IR783-amide **2** as a green powder. (15.8 mg, purity 99.5 %). ^1^H NMR (500 MHz, DMSO-d6): δ 9.56 (s, 1H), 8.96 (s, 1H), 8.12 (d, 2H, J = 5.5 Hz), 7.43 (ABq, 4H, J = 7.0, 5.0 Hz), 7.38-7.32 (m, 4H), 7.16 (t, 2H, J = 7.5 Hz), 7.02 (d, 2H, J = 14.0 Hz), 6.22 (d, 2H, J = 14.0 Hz), 4.05 (bs, 5H), 3.69 (q, 2H, J = 6.0 Hz), 3.38 (q, 2H, J = 6.0 Hz), 2.91 (d, 6H, J = 5.5 Hz), 2.70 (m, 4H), 2.48-2.45 (m, 4H), 1.95 (bs, 2H), 1.70-1.55 (m, 8H), 1.13(s, 12H); ^13^C NMR (125 MHz, DMSO-d6): 170.8, 166.1, 159.9, 158.3, 158.0, 146.3, 142.3, 142.0, 140.3, 133.2, 131.0, 129.4, 128.5, 127.5, 124.6, 122.4, 116.7, 114.4, 11.2, 100.2, 55.8, 50.7, 48.4, 43.7, 42.6, 40.0, 34.9, 27.0, 26.0, 24,2, 22.3, 20.8; HRMS (ESI)^-^: m/z calculated for [M]^+^: C_49_H_63_N_4_O_7_S_2_: 883.8765; found: 883.8768; [M+2H]^2+^: [C_49_H_65_N_4_O_7_S_2_]^2+^: 442.4018, found: 442.4020

***(((2-((E)-6'-((E)-2-(3,3-dimethyl-1-(4-sulfobutyl)-3H-indol-1-ium-2-yl)vinyl)-2'-(2-((E)-3,3-dime-thyl-1-(4-sulfobutyl)indolin-2-ylidene)ethylidene)-2',3',4',5'-tetrahydro-[1,1'-biphenyl]-4-car-boxamido)ethan-1-ylium-1-yl)dimethyl-(4-azanyl)methyl)trifluoroborate (3):*** To a magnetically stirred solution of IR783-amide **2** (20 mg, 22 µmol) in dry DMF (1mL), N, N-diisopropylethylamine (4.0 µL, 22 µmol) followed by 2- (bromomethyl)-4,4,5,5-tetraphenyl-1,3,2-dioxaborolane (9.0 mg, 44 µmol) was added. The reaction was allowed to proceed at room temperature for 4 h. The consumption of IR783-amide **2** and formation of an alkylated product was confirmed by UPLC-MS. Without further purification, 1 M potassium hydrogen fluoride (KHF2, 20 µL) and 3M hydrochloric acid (HCl, 10 µL) were added to the stirring, crude reaction mixture at 0 °C. The reaction was warmed to room temperature over a 1 h period. The resulting solution was quenched with conc NH_4_OH (5 µL), filtered, and diluted with DMF (2 mL). The filtrate obtained was purified by the reverse phase HPLC, using a H_2_O/ACN (0.05% TFA), 40 min elution gradient at a flow rate of 12 mL/min. A linear gradient of increasing ACN from 10% to 60% between 0 and 30 min, followed by a linear increase of ACN from 60% to 90% between 30 and 40 min, were used to elute **3**. The fraction containing **3** was collected and lyophilized to give green solid (11 mg, 13%). ^1^H NMR (500 MHz, DMSO-d6): δ 8.19 (s, 2H), 7.48 (d, 2H, J = 7.0 Hz), 7.43 (d, 2H, J = 8.0 Hz), 7.31-7.39 (m, 4H), 7.15 (t, 2H, J = 7.5 Hz), 7.01 (d, 2H, J = 14.0 Hz), 6.27 (d, 2H, J = 14.0 Hz), 4.12 (bs, 4.0H), 3.71(bs, 4H), 2.48-2.45 (m, 4H), 1.96 (bs, 2H), 1.76-1.65 (m, 8H), 1.09(s, 12H); ^13^C NMR (125 MHz, DMSO-d6): 171.0, 165.6, 159.9, 146.6, 142.1, 140.5, 140.4, 133.0, 130.8, 130.7, 129.4, 128.4, 127.3, 124.5, 122.3, 111.1, 100.2, 63.9, 52.8, 52.6, 48.3, 48.2, 43.5, 34.1, 27.0, 26.0, 24.1, 22.4; HRMS (ESI)^+^: m/z calculated for [M]: C_50_H_64_BF_3_N_4_O_7_S_2_^+^: 964.4261; [M+H]^+^:[C_50_H_65_BF_3_N_4_O_7_S_2_]^-^: 965.6341, found: 965.6899; [M-F]^+^: [C_50_H_65_BF_2_N_4_O_7_S_2_]^+^: 945.8122, found: 945.8105.

# **3. Proof of synthesis**

## 3.1 Characterization of (1)

### 3.1.1 ^1^H NMR (DMSO-d_6_, 500 MHz, 21 °C)


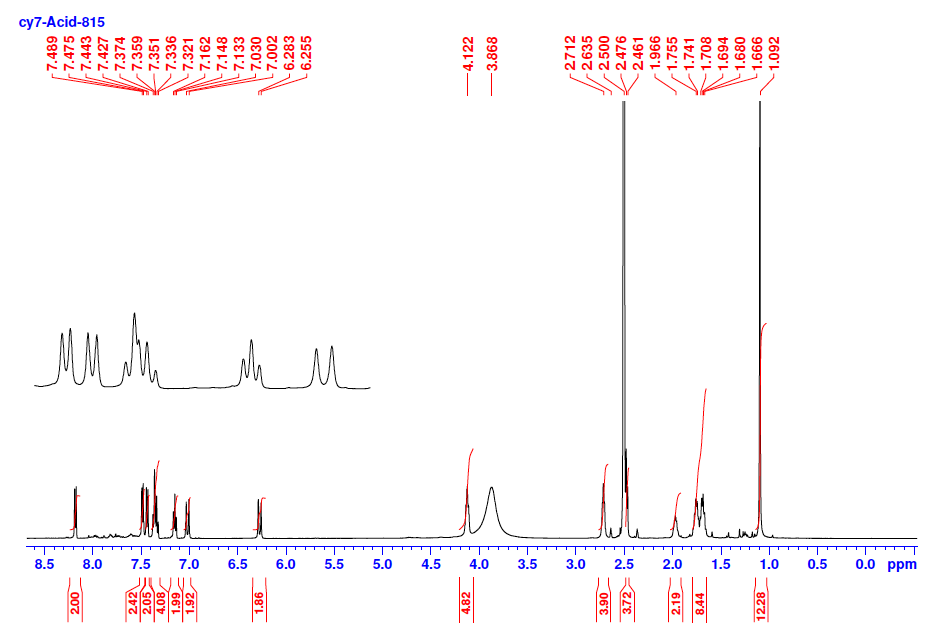


### 3.1.2 ^13^C NMR (DMSO-d_6_, 125 MHz, 21 °C)


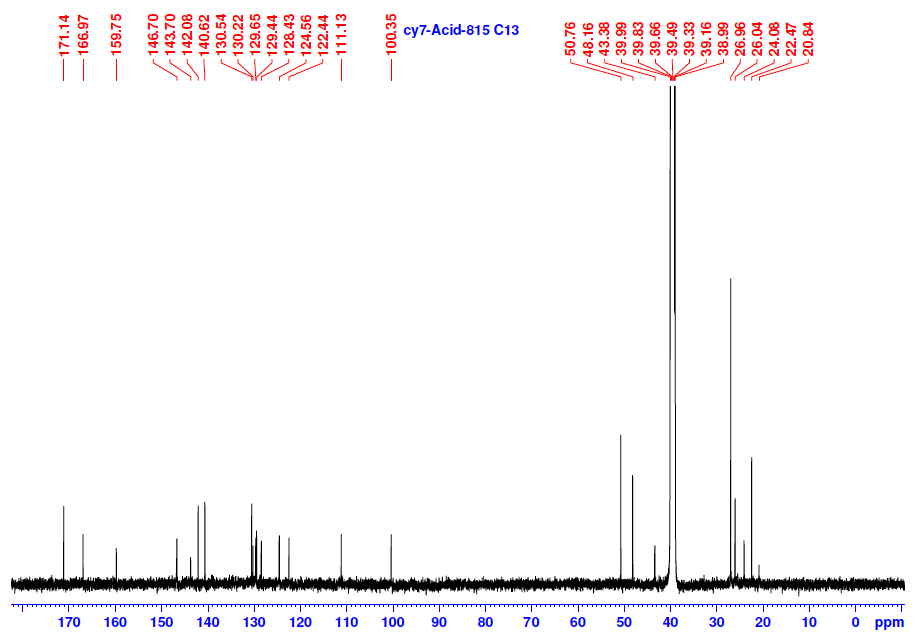


### 3.1.3 HRMS [M]^+^

HRMS (ESI): m/z calculated for [M]^+^: C_45_H_53_N_2_O_8_S_2_^+^: 813.3243; found: 813.3761.

**
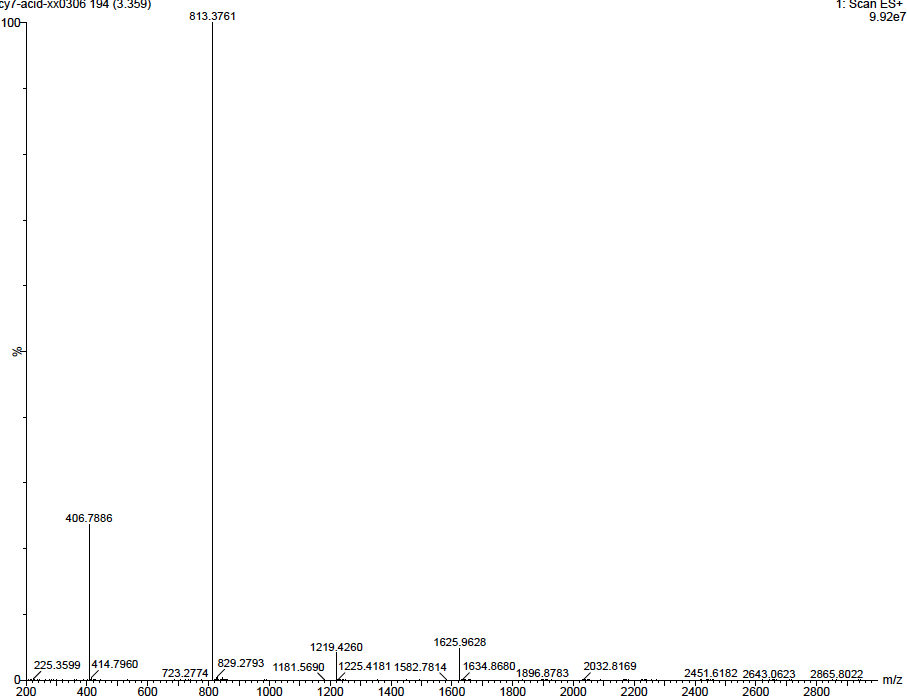
**

## 3.2 Characterization of (2)

### 3.2.1 ^1^H NMR (DMSO-d_6_, 500 MHz, 21 °C)


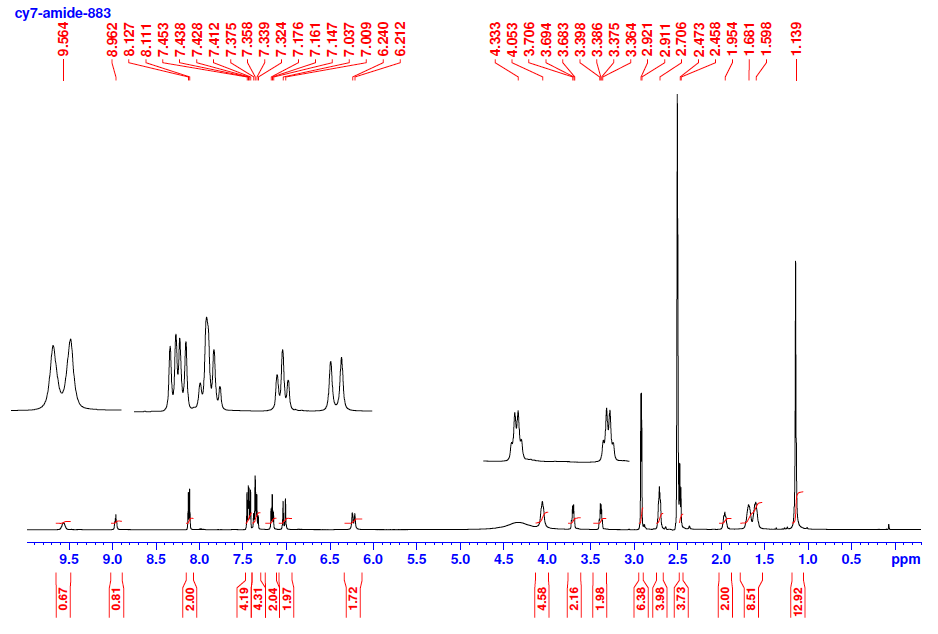


### 3.2.2 ^13^C NMR (DMSO-d_6_, 125 MHz, 21 °C)


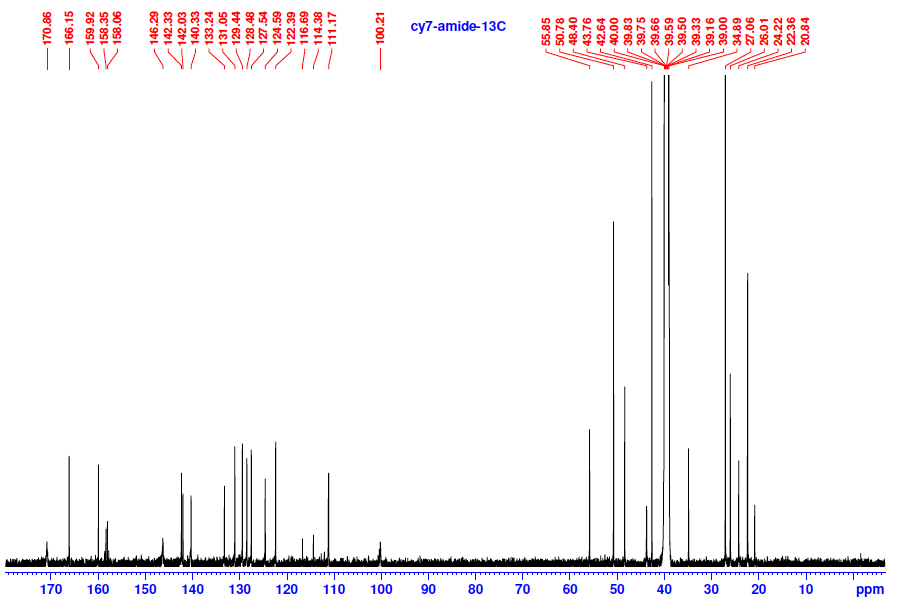


### 3.2.3 HRMS [M+H]^+^

m/z calculated for [M]^+^: C_49_H_63_N_4_O_7_S_2_: 883.8765; found: 883.8768; [M+2H]^2+^: [C_49_H_65_N_4_O_7_S_2_]^2+^: 442.4018, found: 442.4020.


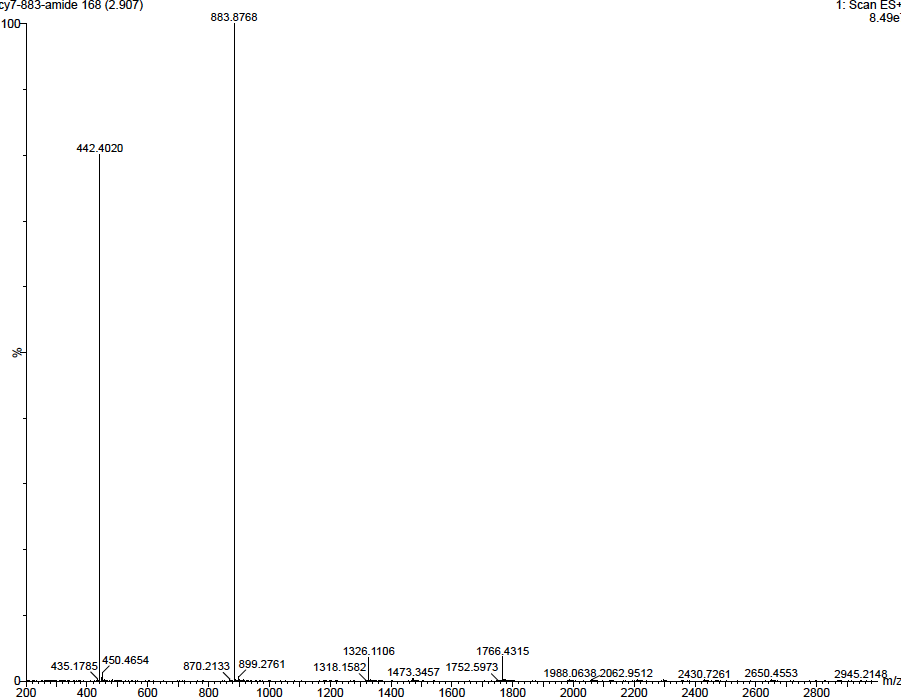


## 3.3 Characterization of (3)

### 3.3.1 ^1^H NMR (DMSO-d_6_, 500 MHz, 21 °C)


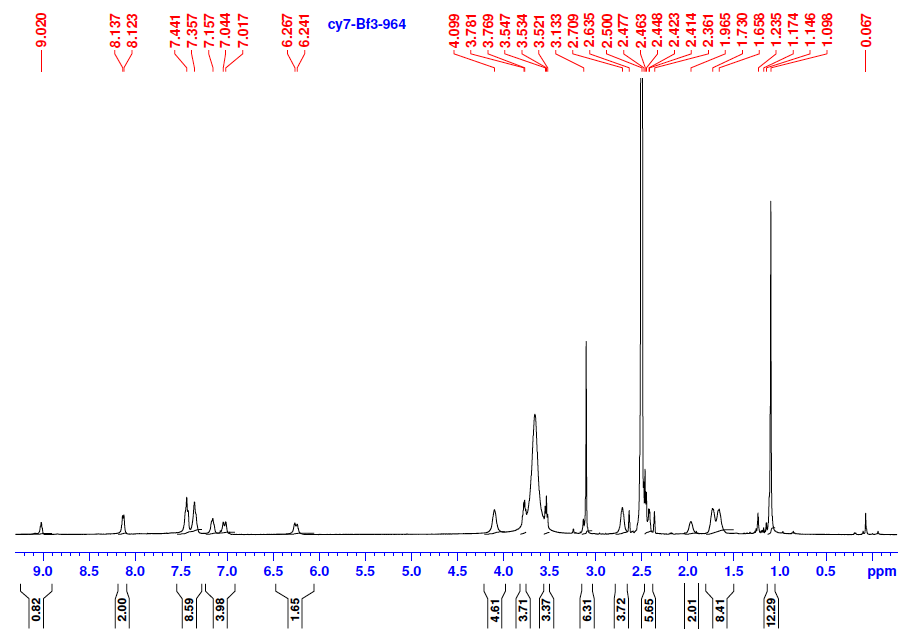


### 3.3.2 ^13^C NMR (DMSO-d_6_, 125 MHz, 21 °C)


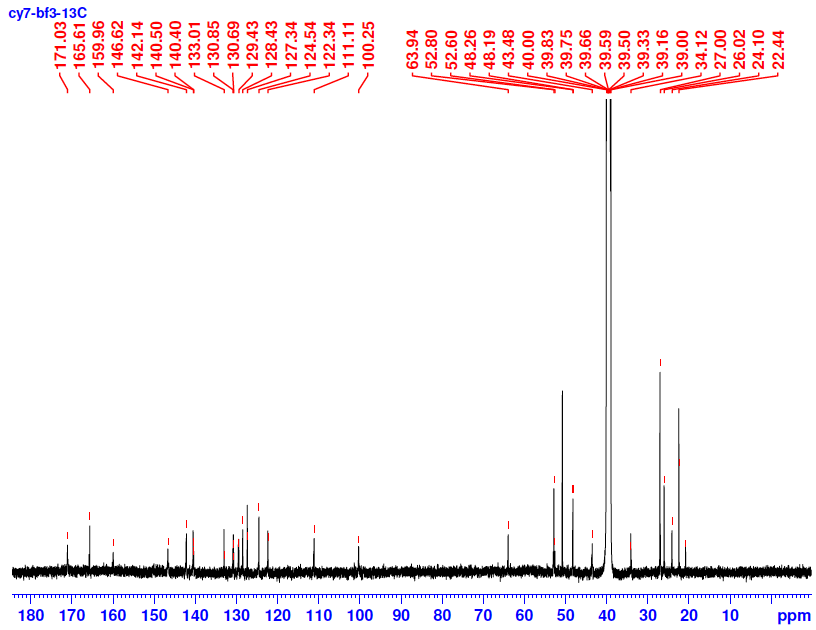


### 3.3.3 ^19^F NMR (DMSO-d_6_, 470 MHz, 21 °C)


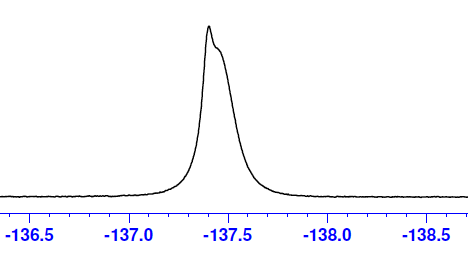

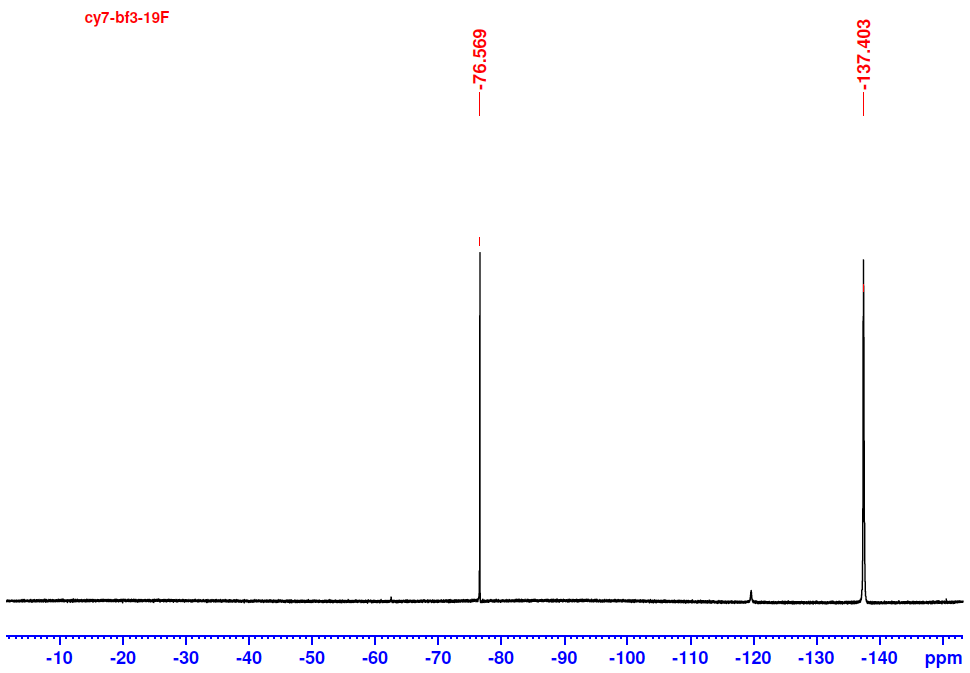


### 3.3.4 UPLC-MS Chromatogram


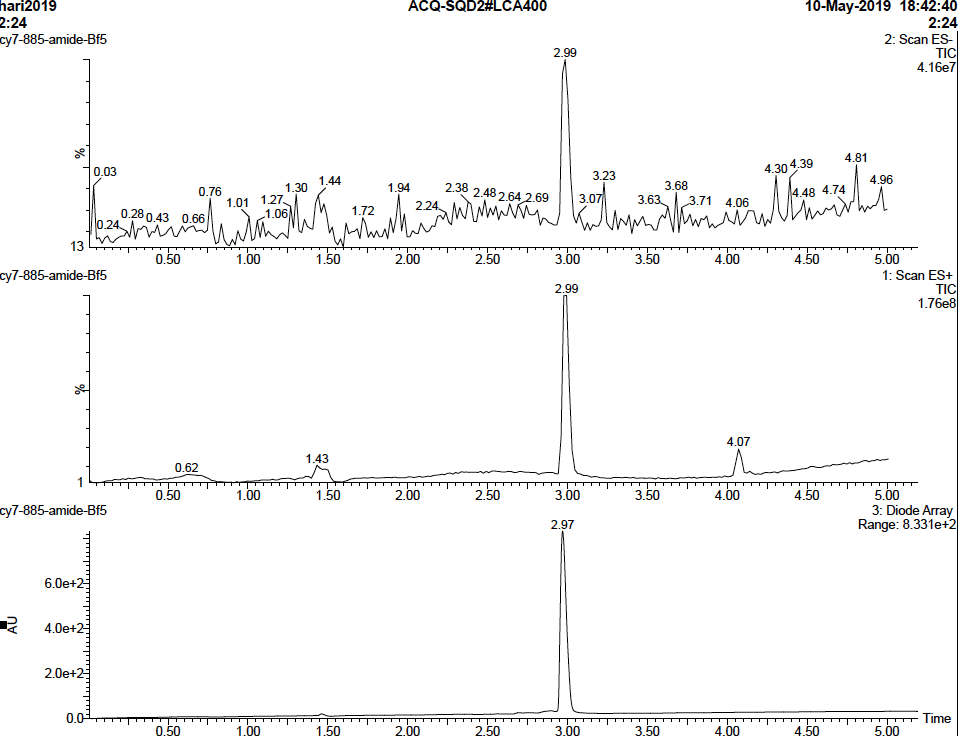


### 3.3.5 HRMS [M+H]^+^, [M-F]^+^

HRMS (ESI)^+^: m/z calculated for (**3**), [M]^+^: C_50_H_64_BF_3_N_4_O_7_S_2_^+^: 964.4261; [M+H]^+^:[C_50_H_65_BF_3_N_4_O_7_S_2_]^-^: 965.6341, found: 965.6899; [M-F]^+^: [C_50_H_65_BF_2_N_4_O_7_S_2_]^+^: 945.8122, found: 945.8105.


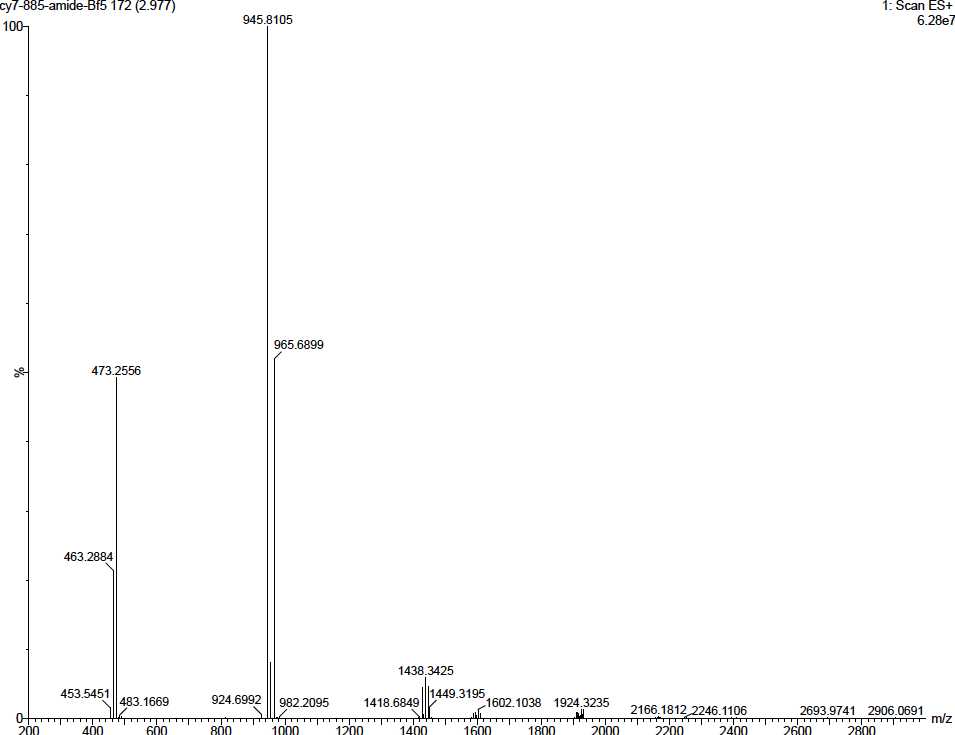


# **4. Fluorescence properties of (IR-783, IR783-AMBF_3_)**

[^19^F]-IR783-AMBF_3_ (3) is fluorescent. The normalized excitation and emission spectra of [^19^F]-IR783-AMBF_3_ and IR-783 (reference) measured in PBS (1 mM, pH 7.4), and EtOH are shown below and in table 1.

### 4.1 Fluorescence spectrum of IR-783 in absolute ethanol

## 4.2 Fluorescence spectrum of (IR783-AMBF_3_) in absolute ethanol

## 4.3 Fluorescence spectrum of IR-783 in 1× PBS, pH 7.4

## 4.4 Fluorescence spectrum of (IR783-AMBF_3_) in 1× PBS, pH 7.4

**4.5 Reverse-phase HPLC of radiolabeled [^18^F]-IR783-AMBF_3_**

**
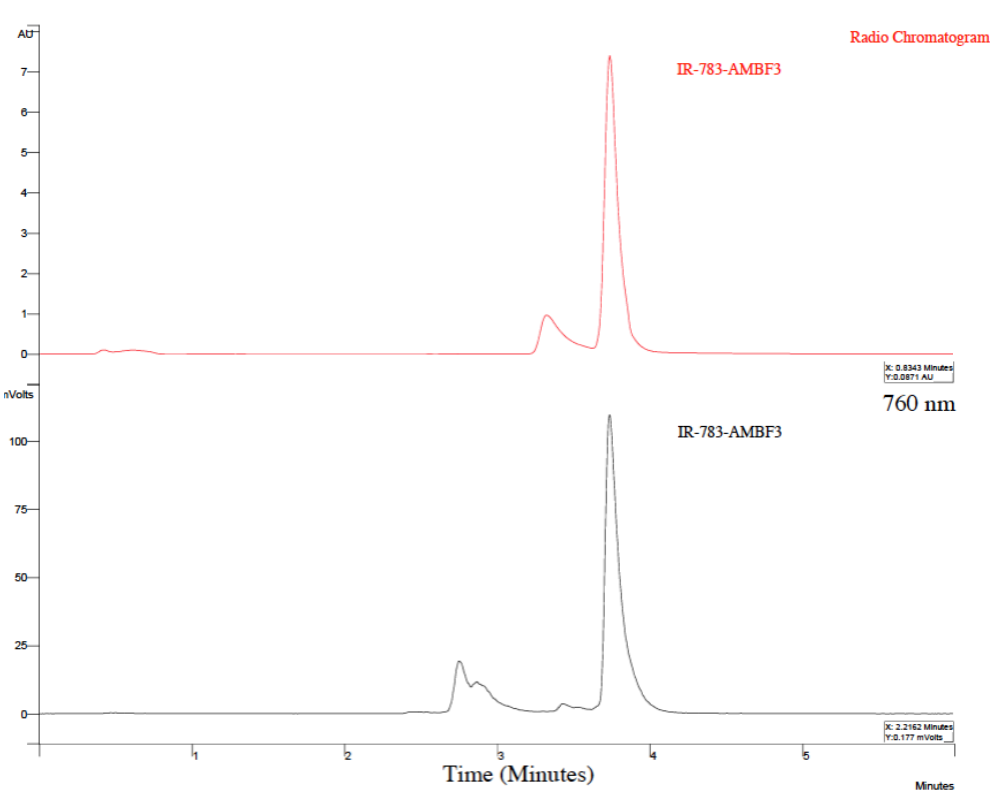
**

# **5. Supporting Table 1. Optical properties of IR783-AMBF_3_ were identified alongside IR-783 in pH 7.4 (1× PBS) and in absolute ethanol.**

|  | Buffer | λ_max_ (nm)  (1-2 µM Solution) | ε (M^-1^ cm^-1^)  (1-2 µM solution) | Excitation (nm) | Emission (nm) | Stokes shift (nm) | Quantum yield (ϕ) |
| --- | --- | --- | --- | --- | --- | --- | --- |
| IR-783 | 1× PBS | 780 | 179,298 | 780 | 802 | 22 | 0.186 |
| IR783-AMBF_3_ | 1× PBS | 778 | 195, 920 | 780 | 793 | 15 | 0.183 |
| IR-783 | EtOH | 790 | 179,980 | 780 | 802 | 12 | 0.084 |
| IR783-AMBF_3_ | EtOH | 775 | 178,332 | 780 | 787 | 12 | 0.085 |

**6. Supporting materials and methods**

**6.1 Cytotoxicity assessment**

9L/lacZ, bEnd.3 or HMEC-1 cells at a density of 5000 cells/well were seeded in the 96-well plates and incubated overnight for adhesion. Cells were treated with different concentrations of IR783-AMBF_3_ up to 50 µM and incubated for 24 h at 37 °C. IR-783 was used as control. Following incubation, the cells were rinsed by 1× PBS, and 100 µL culture medium containing 10 µL MTS reagent (Promega, G3581, WI, USA) was added to the plates. Following incubation for 1.5 h at 37 °C, 100 µL was collected and its absorbance was measured at 490 nm on a microplate reader (Infinite M1000 Pro, Tecan, USA).

**6.2 Tissue distribution**

Anesthetized rats received intrathecal catheterization (PE10) as previously described. 100 µCi of IR783-AMBF_3_ was injected into the CSF space of rats through the catheter over a 1 min period. Rats were euthanized by CO_2_ inhalation overdose at 2 h and 4 h post-injection (n = 3). Main organs were harvested, weighted, and counted on a Wallac Wizard 3.0 gamma counter. Tissue radioactivity levels were presented as percentage injected dose (%ID) per gram of tissue.

**6.3 *In vivo* toxicity assessment**

Anesthetized rats with intrathecal catheter (PE10) were infused with 150 µM IR783-AMBF_3_ (300 µL, 15-fold higher than the imaging dose) through the lumbar catheter at a rate of 10 µL/min (n = 4). The control group of rats was intrathecally injected with PBS (300 µL). The rats were weighed and monitored daily for up to 32 days.

**6.4 Histological analysis**

Rats were intrathecally injected with 10 µM IR783-AMBF_3_ (300 µL) through a lumbar catheter and euthanized at 20 min post-injection. Then the brain was collected and fixed in 4% paraformaldehyde/PBS for 6 h. Following fixation, the brain was embedded into Optimal Cutting Temperature (OCT) medium (Sakura Tissue-Tec, #4585). 20 µm-coronal sections were created on a Bright OTF 5000 cryostat (Bright Instruments, UK). The samples were mounted with medium containing DAPI (Vector Laboratories, USA), and fluorescent images were taken using the EVOS microscope (Life Technologies, USA) at an excitation/emission of 710/775 nm.

**6.5 Lumboperitoneal shunt**

One end of a PE60 shunt (0.76 mm I.D., 1.22 mm O.D., Instech Laboratories Inc., Plymouth Meeting, PA) was inserted into the subarachnoid space between L5 and L6 as described previously. The PE60 had to be heated by a lighter and then stretched out to obtain a smaller diameter (about half of the diameter) at the lumbar end to perform catheterization. Then the distal end of shunt was tunneled under the skin and threaded through the peritoneal cavity through a small incision placed in the skin and abdominal muscles. Before a suture was placed in this incision, the shunt was filled with 10 µM IR783-AMBF_3_ (300 µL) and the peritoneal end of the shunt was placed into peritoneal cavity. Following the shunt placement, fluorescence images were obtained on each rat at different time points up to 24 h on the Bruker In-Vivo imager. Fc-AMBF_3_ was used as a control.

**7. Supporting Figures**


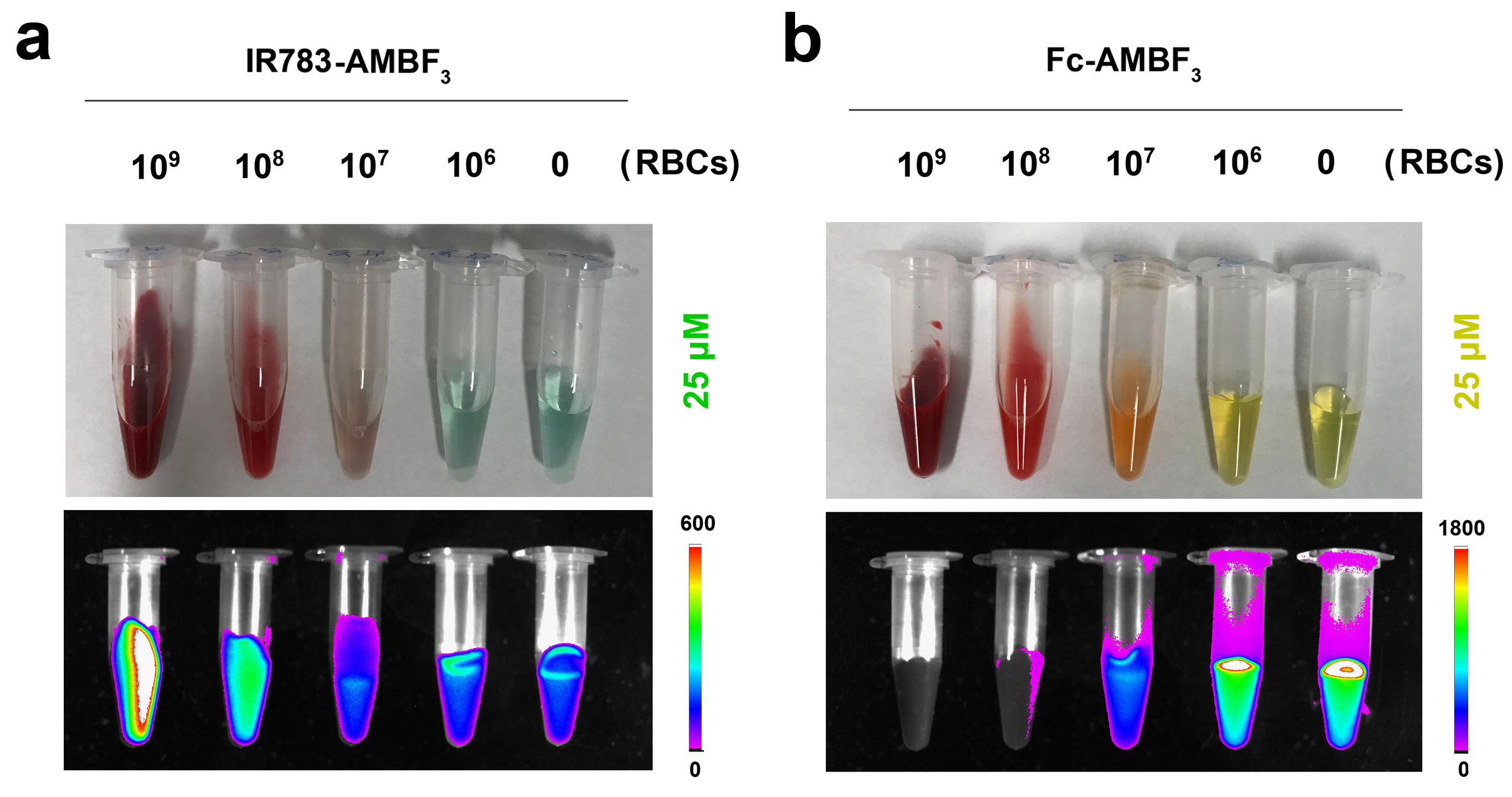


**Fig. S1.** Fluorescence imaging of static concentrations of IR783-AMBF_3_ (a) and Fc-AMBF_3_ (fluorescein, 25 µM) (b) when mixed with an increasing number of RBCs. Fc-AMBF_3_ fluorescence is reduced in the presence of RBC, while minimal fluorescent signal perturbation is observed in IR783-AMBF_3_-RBC mixtures. IR783-AMBF_3_ is a superior tool for imaging CSF leakage in volumes and cavities that are contaminated with blood. Excitation and emission filters for IR783-AMBF_3_ and Fc-AMBF_3_ are 760/830 nm and 450/535 nm respectively. The exposure time is 1 sec. Fluorescence data was collected on a Bruker extreme.


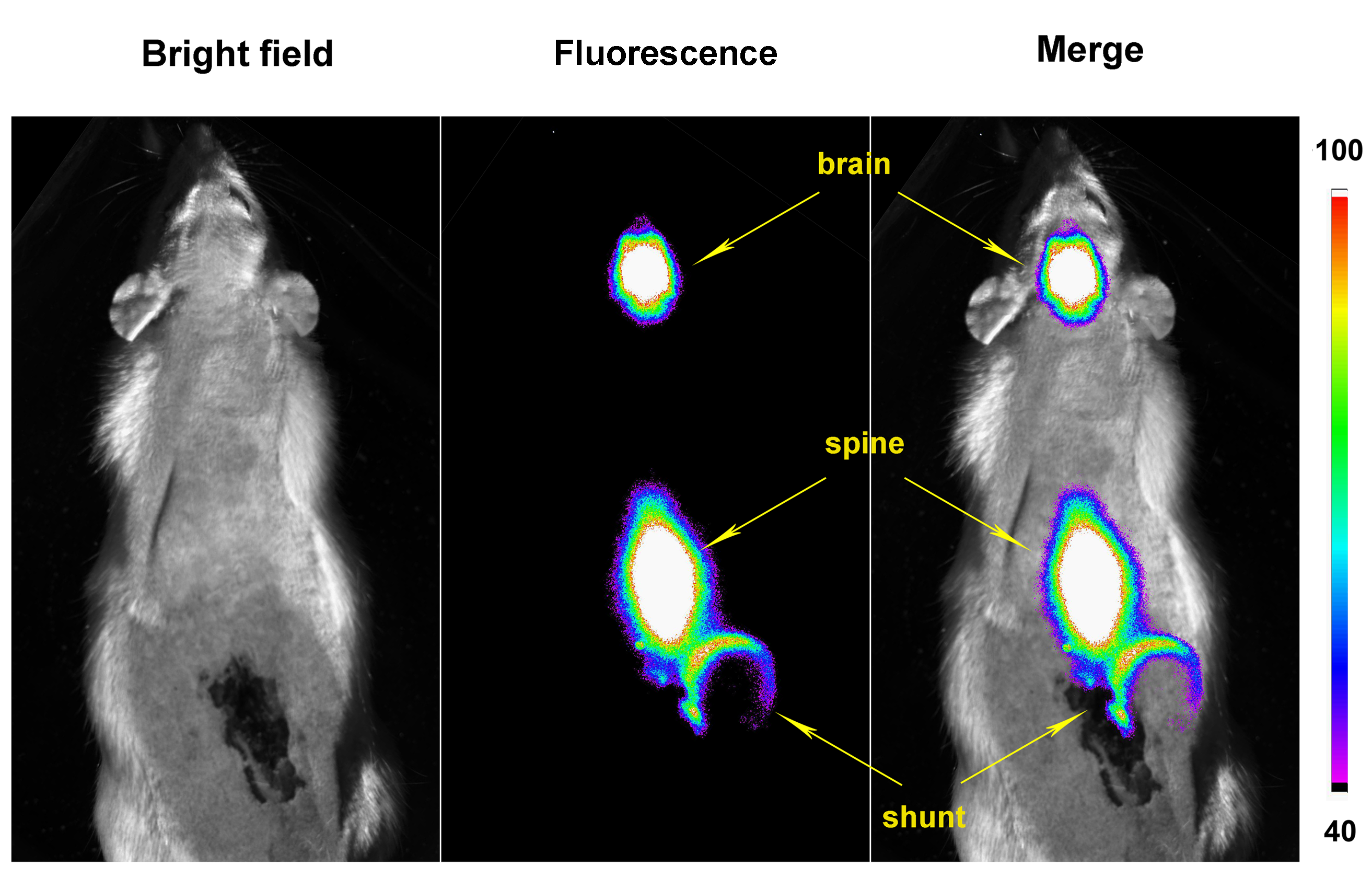


**Fig. S2.** IR783-AMBF_3_ allows for deep tissue fluorescence imaging through the skin, in the spine, in the cisterna magna, and in lumbar-peritoneal shunts in a live (shaved) rat. The *in vivo* imaging of IR783-AMBF_3_ immediately after intrathecal injection into a rat bearing a subcutaneous catheter. IR783-AMBF_3_ in a catheter implanted superficially under the skin is clearly visible (exposure time: 20 sec). IR783-AMBF_3_ fluorescence is visible in the shunt through the skin, indicating a viable shunt.


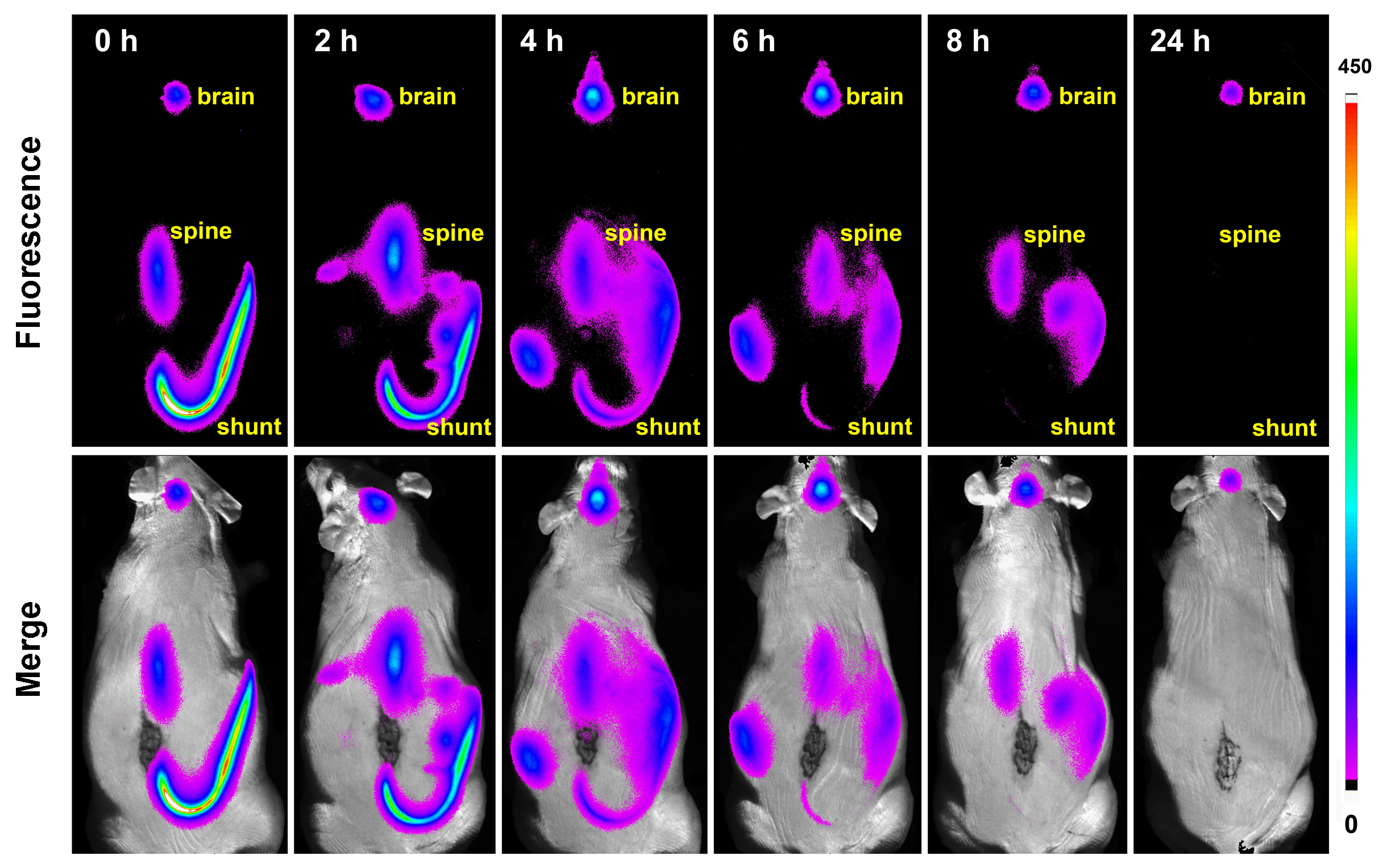


**Fig. S3.** Real time imaging of intrathecally introduced (Lumbar 5-6) IR783-AMBF_3_ on a rat. IR783-AMBF3 clears from the CSF containing spine (0–4 h) through a viable LP shunt (0–4 h) into the intraperitoneal space (2–8 h, Ex/Em: 790/830 nm, exposure time: 20 sec). Superficial IR783-AMBF_3_ fluorescence can be seen through shaved skin; in the shunt, spine, and brain; and through the skin as it is cleared through the viable shunt into the peritoneal cavity.
